# New genes drive the evolution of gene interaction networks in the human and mouse genomes

**DOI:** 10.1186/s13059-015-0772-4

**Published:** 2015-10-01

**Authors:** Wenyu Zhang, Patrick Landback, Andrea R. Gschwend, Bairong Shen, Manyuan Long

**Affiliations:** Center for Systems Biology, Soochow University, Suzhou, Jiangsu 215006 China; Department of Ecology and Evolution, The University of Chicago, Chicago, IL 60637 USA; Committee on Genetics, The University of Chicago, Chicago, IL 60637 USA; Department of Bioinformatics, Medical College, Soochow University, Suzhou, Jiangsu 215123 China

## Abstract

**Background:**

The origin of new genes with novel functions creates genetic and phenotypic diversity in organisms. To acquire functional roles, new genes must integrate into ancestral gene-gene interaction (GGI) networks. The mechanisms by which new genes are integrated into ancestral networks, and their evolutionary significance, are yet to be characterized. Herein, we present a study investigating the rates and patterns of new gene-driven evolution of GGI networks in the human and mouse genomes.

**Results:**

We examine the network topological and functional evolution of new genes that originated at various stages in the human and mouse lineages by constructing and analyzing three different GGI datasets. We find a large number of new genes integrated into GGI networks throughout vertebrate evolution. These genes experienced a gradual integration process into GGI networks, starting on the network periphery and gradually becoming highly connected hubs, and acquiring pleiotropic and essential functions. We identify a few human lineage-specific hub genes that have evolved brain development-related functions. Finally, we explore the possible underlying mechanisms driving the GGI network evolution and the observed patterns of new gene integration process.

**Conclusions:**

Our results unveil a remarkable network topological integration process of new genes: over 5000 new genes were integrated into the ancestral GGI networks of human and mouse; new genes gradually acquire increasing number of gene partners; some human-specific genes evolved into hub structure with critical phenotypic effects. Our data cast new conceptual insights into the evolution of genetic networks.

**Electronic supplementary material:**

The online version of this article (doi:10.1186/s13059-015-0772-4) contains supplementary material, which is available to authorized users.

## Background

New genes provide important genetic novelties responsible for biological diversity in organisms [1], and are often the genetic basis for lineage- or species-specific components in important biological processes and structures [2, 3]. As biological characteristics mostly emerge through complicated interactions among a cell’s components [4], new genes will inevitably be integrated into and reshape ancestral gene-gene interaction (GGI) networks to acquire their corresponding biological roles. Recently, several case-studies have shown individual new genes can participate in local ancestral GGI networks and acquire important functions in fruit fly [5, 6], budding yeast [7], and plants [8, 9]. Consequently, it is intriguing to ask how new genes are topological and functionally incorporated in and subsequently change ancestral GGI networks in genome-wild scale.

Thanks to the accumulation of GGI data brought by the development of high throughput technologies, a couple of attempts have been made to address this issue. Through examining the evolution of new genes in the protein-protein interaction networks of yeast Saccharomyces cerevisiae, Capra *et al.* [10] found novel genes are less integrated in cellular networks than duplicated genes, genes prefer to interact with other genes of similar age and origin, and new genes participated in the network modules for synthesis of important metabolites. By applying different network data source, another research group showed a similar integration process of new genes in yeast [11]. Popadin *et al.* [12] recently analyzed a co-expression network with previous data of gene ages in vertebrates [2, 13] and observed a difference of integration of these genes into the networks between young and old ages. These works encourage us to further explore a potential quantitative correlation between a continuous evolutionary process of new genes and their degree to be integrated into and subsequent rewiring of various ancestral gene networks in vertebrates, which have provided data of evolutionarily well resolved divergence times and interesting phenotypic data with the rich datasets of recently evolved genes we identified [2, 13].

In the present report, we investigated evolutionary patterns of GGI networks driven by new genes originating throughout various stages in the lineages toward human and mouse. Taking advantage of a well-resolved gene dating dataset [2, 13] and the rich and independent GGI datasets, we elaborately explored the integration process of new genes into GGI networks reconstructed with four different data sources in both human and mouse. Following, we focused on the functional evolution analysis of new genes in human genome, and explored how new genes acquire critical functions, that is, pleotropic functions, essential functions, and brain development relevant functions, in term of GGI network integration. Finally, we deeply excavated and discussed the mechanisms driving the evolution of GGI networks and deriving the integration patterns of new originating genes.

## Results and discussion

### The integration of new genes into GGI networks is a gradual evolutionary process

A technical challenge to examine the role of new genes in evolution of gene networks is to detect reliable GGI networks in their global distribution. Considering current technical growth and evaluation to methods and data that reveal GGI, we constructed and analyzed three different types of data in attempt to identify robust GGI networks (see Methods): the human protein-protein interactions (hPPIs), the human gene co-expression (hGC) networks, and the mouse protein-protein interactions (mPPIs).

The second line of data we used to investigate the correlation between new gene evolution, as we extensively investigated previously, and the evolution of GGI networks as revealed by above three different databases is the best-resolved vertebrate divergence times, supported by paleontology, organismal evolutionary analysis, and molecular evolution, and most reliably resolved phylogenetic tree of vertebrates over decades of extensive studies on vertebrate species [2, 13]. These data provided excellent estimates for the ages of new genes, comprising the ones generated by DNA-based duplication, RNA-based duplication, and *de novo* origination during the vertebrate evolution in the lineage toward humans and mouse, as we identified previously in comparative genome comparison.

First of all, we investigated the correlation between the ages of genes and their topological characteristics in the GGI networks described in the four databases we constructed. Remarkably, all these types of GGI network data revealed highly similar rates and patterns of new genes-integrated into the networks. Therefore, we will focus on human for presentation and discussion of the results while introducing the relevant findings in the mouse genome.

**Fig. 1:**
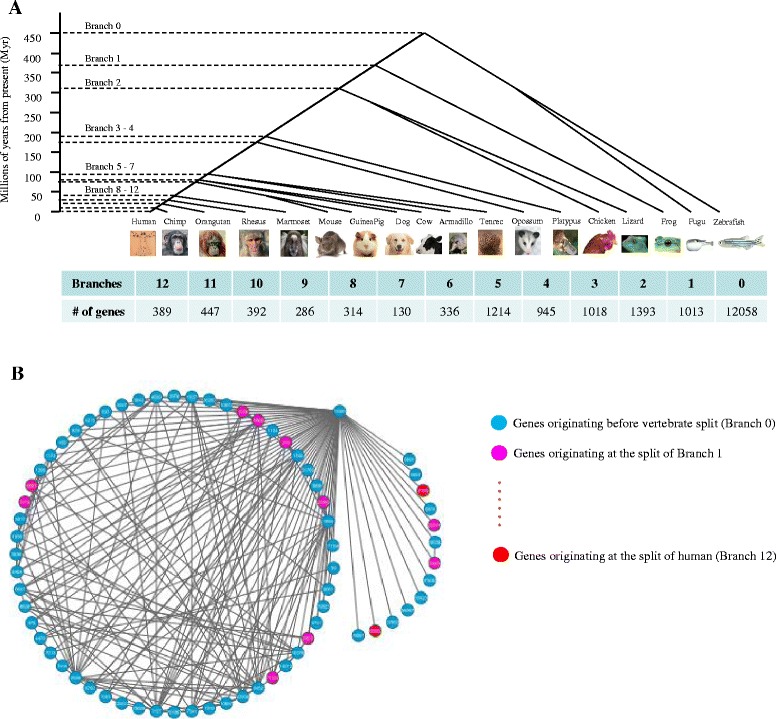
Schematic diagram to show the network integration of new genes originating from various phylogenetic branches towards human. **a** Phylogenetic tree of vertebrates towards human together with branches and divergence times in millions of years from present (myr). The number of genes originating at each phylogenetic branches was also listed. **b** A sub-graph of human PPI network to show the incorporation of new genes from different originating times

**Fig. 2:**
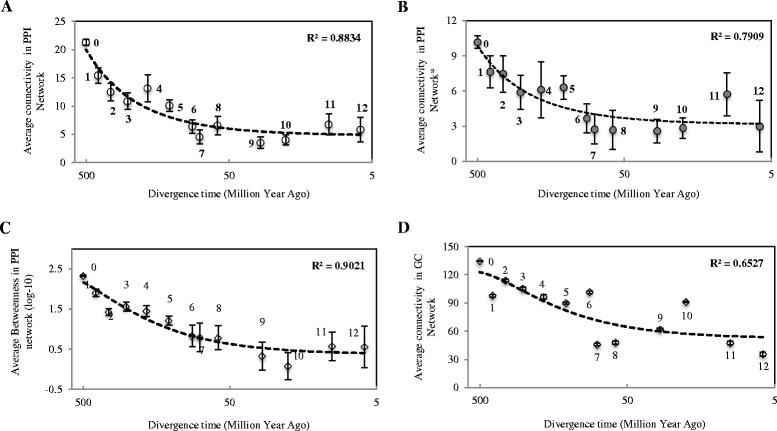
GGI network topological patterns of human genes related to their divergence times. **a** Distribution of PPI network connectivity (numbers of interactions) for genes from different phylogenetic branches. **b** Distribution of genes from different phylogenetic branches from another PPI network connectivity reconstructed with a more stringent threshold. **c** Distribution of average Betweenness (log10-based) within each gene group in the PPI network. **d** Distribution of GC (gene co-expression) network connectivity for genes from different phylogenetic branches. The error bars show the standard error of the mean for each group of genes, and the dash line indicates the polynomial regression correlation between network centralities (that is, Connectivity, Betweenness) of genes and their divergence times. Numbers near each data point are phylogenetic branch assignments for each group of genes. The divergence time of each gene age group was assigned as the middle time point for each branch and the oldest branch (branch 0) is arbitrarily set as 500 myr

We first analyzed the human protein-protein interactions (hPPIs) network by exploiting and modifying an integrative experimental protein interactions dataset [14] (with the threshold of confidence score of 0.68, see Methods). The reconstructed human PPI network revealed an approximately scale-free topological structure [15] with a degree exponent of 1.49 that defines a power-law distribution of connectivity (or degrees) (Additional file 1: Figure S1 and Additional file 2: Table S1). We then labeled the gene (equivalent to its coded protein) age of each node in the PPI network, determined by an age index for the genes that originated in every period of evolution along the well-resolved phylogeny of vertebrates (Fig. 1a and b), that were retrieved from a widely used database [2, 13] (See Methods). Analysis on the above PPI network indicated a significant and strong correlation (Polynomial regression test, *R*^*2*^ = 0.8834, Fig. 2a) between the ages of genes and their connectivity (or degree, that is, numbers of interacting partners) in the PPI network, revealing a gradual evolutionary process in which new genes are integrated into the PPI network, which echoed the evolutionary procedure of new gene structures [16]. This finding suggests that throughout vertebrate evolution there was a non-robust and rapid process, unexpected by conventional thought, in which new genes were integrated into the GGI networks. During this process of 370 million years (MY, branch 1–12, Fig. 1a) we examined, we observed that 5,710 new genes were integrated into the GGI networks. Furthermore, this process showed an evolutionarily significant pattern: the new genes started, at a young age, to be integrated into networks to form new and less connected branches; however, with the elapse of evolutionary time, as genes grow older, they acquired more interacting links.

To avoid possible bias created by the chosen confidence score threshold for the reconstruction of human PPI network, we reanalyzed a new human PPI network using a more stringent cutoff (With minimum confidence score of 0.77, see Methods and Additional file 2: Table S1) and we found the same evolutionary pattern (Polynomial regression test, *R*^*2*^ = 0.7909, Fig. 2b). The connectivity-based conclusion is further supported by the analysis of another statistic parameter describing network centralities of genes, that is, Betweenness, which measured the importance of one node connecting all the other nodes (Polynomial regression test, *R*^*2*^ = 0.9021, Fig. 2c). Based on human PPI network reconstructed from a different experimental manual curation resource (See Methods and Additional file 3: Figure S2A), that is, Human Protein Reference Database (HPRD) [17], the same conclusion was drawn as described above (Additional file 3: Figure S2B).

For a more rigorous analysis of independent GGI data types, we analyzed another human GGI network referred to as gene co-expression (hGC) network (See Methods and Additional file 3: Figure S2C and D), reflecting the correlations of gene expression profiling in a series of human tissues [18]. Mapping the topological positions of new genes in humans into the GC network revealed a similar correlation between the ages and connectivity of genes (Polynomial regression test, *R*^*2*^ = 0.6527, Fig. 2d), revealing the same evolutionary trend of new genes starting with low connectivity and evolving to be highly connected hubs. Additionally, we also explored the evolutionary patterns of human PPI network based on another gene age dataset [19] (Additional file 4: Figure S3A), which estimated gene ages in human genome based on independent and long distant phylogenetic distribution. A same evolutionary pattern of new genes was shown (Additional file 4: Figure S3B), and it was further demonstrated that our conclusion was independent of gene age dating datasets. Thus, different GGI data, that is, PPI and GC data, and different gene age dating data, all supported the same conclusions as reported above.

Furthermore, we applied a similar protocol to analysis of the reconstructed mouse GGI networks from mouse PPI data (mPPIs), by integrating most of the available online experimental interaction datasets (Additional file 5: Table S2). The integrative analysis of mouse gene age information [13] (Additional file 6: Figure S4A) and PPI topological data (Additional file 6: Figure S4B) lead to the same conclusion (Polynomial regression test, *R*^*2*^ = 0.6232, Additional file 6: Figure S4C) determined by the human GGI network analyses. These data suggest a gradual integration of new genes in the GGI networks is an evolutionary process shared in mammalian lineages of primates and rodents.

**Fig. 3:**
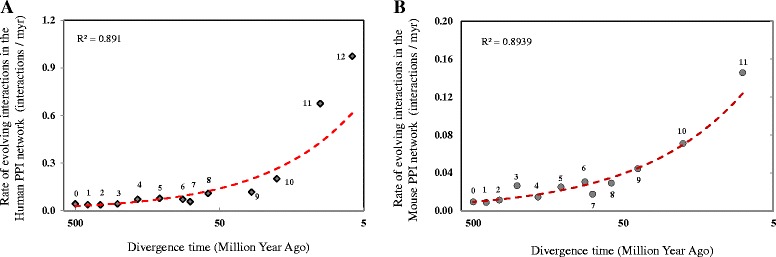
Average rate of evolving linking partners (interactions / myr) for genes from different phylogenetic branches based on the human PPI network (**a**) and mouse PPI network (**b**). The dash line indicates the power regression correlation between evolution rates of interactions for genes and their divergence times. Numbers near each data point are phylogenetic branch assignments for each group of genes. The divergence time of each gene age group is assigned as the middle time point for each branch. And the oldest branch (branch 0) is arbitrarily set as 500 myr

Given the observation that the acquisition of genetic interactions is a time-dependent gradual procedure, we further investigated whether this process occurred at a constant rate. Our result showed that new genes could establish linking partners at a high rate (interactions acquired per million years) in the initial stage of their origination. After that, the rate dramatically declined, and finally plateaued (Fig. 3a and b), suggesting that the acquisition of biological roles of new genes is a rapid process during early evolution, but as the genes age, the function spectrum is diversified at a much lower rate. Taking advantage of the high coverage of the human PPI data (Additional file 2: Table S1), we subsequently focused on the analysis of both topological and functional evolution patterns of new genes based on our first constructed human PPI network.

**Fig. 4:**
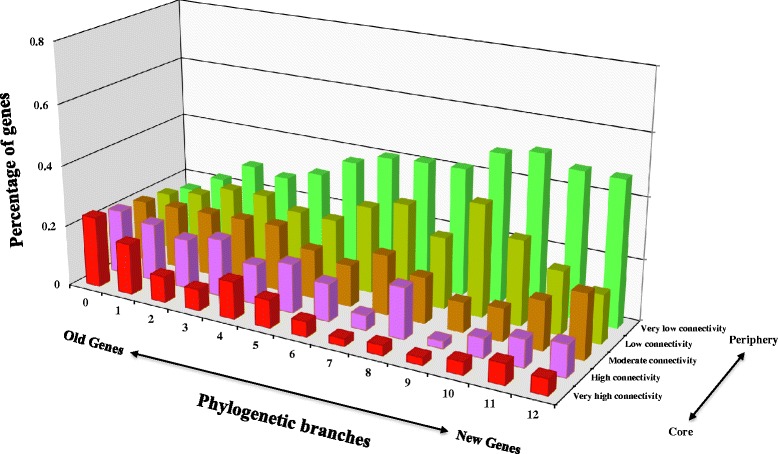
PPI network locations of genes in relation to their divergence times. The network locations of genes are classified into five distinct layers according to the rank of degree centralities. Specifically, genes that have the top 20 % of degree centralities are assigned to the network core (genes with very high connectivity), and those with the bottom 20 % of degree centralities in the network periphery (genes with very low connectivity). The same rule is applied for the assignment of the middle three network layers. The x-axis shows the phylogenetic branches for each group of genes, and y-axis indicates the categorization of genes according to the above specifications, and z-axis exhibits the percentage of genes within each age group located in the corresponding categories

To better visualize the integration process, we mapped the genes in the mammalian GGI networks based on their connectivity, where highly connected genes made up the core of the human PPI network and genes with low connectivity were located on the network periphery (Fig. 4), which revealed a clear correlation between gene age and location in the mammalian GGI networks. Surprisingly, a small fraction of young genes were found to have evolved into the network core, whereas the majority of recently originating genes, especially primate-specific genes (branch 8–12, Fig. 1a), are located in the exterior regions of network. As the ages of genes increase, they tend to appear more frequently in the more densely connected core of network.

### New genes gradually acquire pleiotropic and essential function roles

**Fig. 5:**
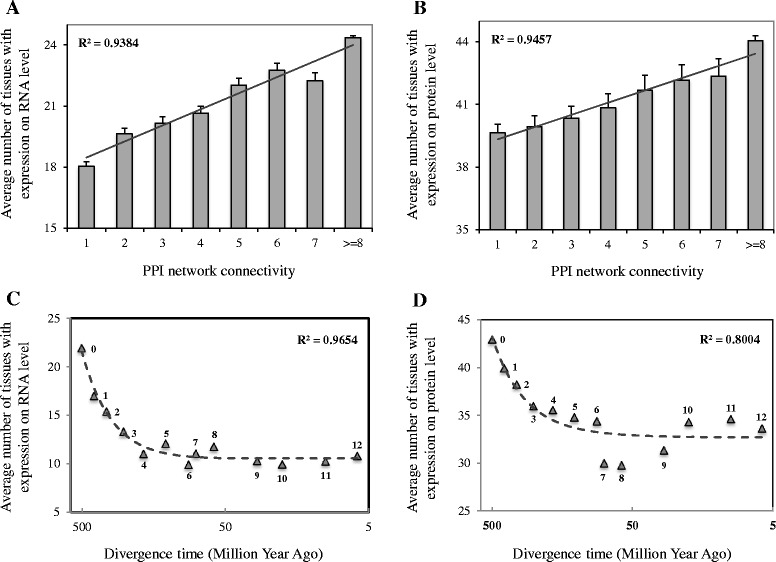
Expression breadths of genes in regards to their PPI network connectivity and divergence times. **a** Average number of tissues with expression of genes with various PPI network connectivity based on RNA-seq expression level data. **b** Average number of tissues with expression of genes with various PPI network connectivity based on protein expression level data. The error bars show the standard error of the mean for each group of genes, and the solid line indicates the linear regression correlation between network connectivity of genes and their expression breadths. **c** Average number of tissues with expression of genes from different phylogenetic branches based on RNA-seq expression level data. **d** Average number of tissues with expression of genes from different phylogenetic branches based on protein expression level data. The dash line indicates the polynomial regression correlation between divergence times of genes and their expression breadths. Branch assignment is labeled near each data point. The age assignment for each branch follows Fig. 1

As most biological characteristics arise from the complex interactions between cell’s numerous components [4], the integration of new genes into the GGI network might indicate the emergence of novel functions for these new genes. Furthermore, the gradual evolution of more interactions in GGI networks might signal the process of new genes acquiring pleiotropic functions. This hypothesis could be indirectly confirmed by the strong correlation of connectivity of genes and their divergence times (Fig. 2a) and a strong linear correlation between the connectivity of genes and their expression breadths at both RNA expression level (Pearson linear correlation test, *R*^*2*^ = 0.9384, Fig. 5a) and protein expression level (Pearson linear correlation test, *R*^*2*^ = 0.9457, Fig. 5b). Thus it could hint that new genes gradually evolve broader expression patterns and therefore acquire pleiotropic functions, as they gradually evolve more linking partners (Fig. 2a), and genes with more linking partners tend to have broader expression patterns (Fig. 5a and b).

To verify this hypothesis in a direct manner, we further computed and compared the tissue expression patterns for genes along different phylogenetic branches. Our results showed that genes gradually evolved broader tissue expression patterns at mRNA expression level from RNA-seq data [20] (Polynomial regression correlation test, *R*^*2*^ = 0.96538, Fig. 5c), which indicates the acquirement of stronger pleiotropic functions. One might dissent the role of mRNA as the performer of biological functions, our analysis on protein expression profiling data [20] drew the same conclusion (Polynomial regression test, *R*^*2*^ = 80038, Fig. 5d). In line with the network topological integration process of new genes (Figs. 2a and 4), our results showed a gradual process for new genes to evolve pleiotropic function roles, reflected by the tissue expression patterns. These findings also suggest functional constraints on new originating genes [21], as they are usually shown to be with very narrow and specified expression patterns [22], such as testis expression [23].

**Fig. 6:**
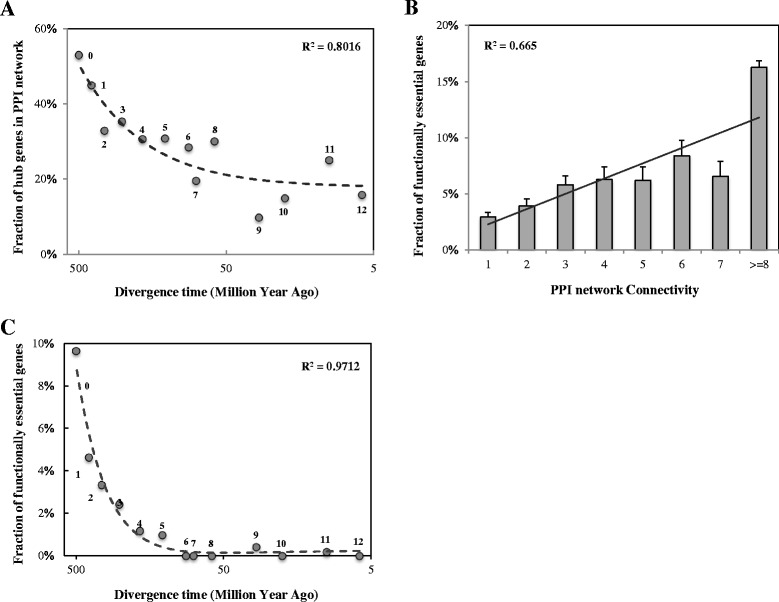
Fraction of topologically and functionally essential genes for gene groups from different divergence times. **a** Fraction of hub genes in PPI network within gene groups of different divergence times. Hub genes are defined as genes with network connectivity greater than median level (Interaction degree > = 6). Branch assignment is labeled near each data point. The age assignment for each branch follows Fig. 1. The dash line indicates the polynomial regression correlation between divergence times of genes and the fractions of hub genes. **b** Fraction of essential genes in regards to their PPI network connectivity. The solid line indicates the linear regression correlation between PPI network connectivity of genes and the fractions of essential genes within each gene group. **c** Fraction of essential genes in PPI network within gene groups from different divergence times. The dash line indicates the polynomial regression correlation between divergence times of genes and the fractions of essential genes

One critical feature of scale-free networks is the existence of hub nodes, or highly connected nodes [24]. Hub nodes are essential components in various networks [25], and are subjected to concentrated evolutionary forces that shape the network structures to result in essential functions [3, 26]. To explore the contribution of new genes in reshaping the GGI network, we investigated the percentage distributions of hub genes (with interaction degrees no smaller than 6) originating across different phylogenetic branches in human PPI network. The data revealed a strong correlation between gene ages and fractions of hub genes (Polynomial regression correlation test, *R*^*2*^ = 0.8016, Fig. 6a). In particular, we found a high proportion of hub genes (16 %) arising in the most recently originated human-specific branch (Branch 12, Fig. 1a), and this number gradually increased with gene ages, peaking at around 53 % for the earliest originating genes (Branch 0, genes arising before the split of vertebrates, Fig. 1a). This phenomenon indicates the gradual process of new genes evolving to be network hubs, and reshaping the original gene interaction networks.

It has been reported that there is a relationship between gene topological features and biological functions [26, 27]. More specifically, genes with high network connectivity tend to be functionally essential [26] (Fig. 6b). Given the above observation that new genes gradually evolve many interactions to become network hubs, it is reasonable to infer that the acquisition of functional essentiality for new genes in human genomes may follow a step-wise evolutionary process. Through the meticulous collection and analysis of sources of human gene essentiality data (Additional file 7: Table S3, see Methods), we explored the relationship between gene essentiality and origination time (Fig. 6c). It was unexpected that a proportion of newly originated genes, especially genes that arose after branch 6 (approximately 80 million years ago), have evolved essential functions, although more genes originating from older periods are functionally essential, and the fraction of essential genes increases with the elapse of evolutionary time. Together with aforementioned observations from the network topology, our analysis demonstrated a clear trend that human new genes gradually evolve to be topologically central and functionally essential, and acquire the capability to reshape the GGI networks.

### Human-specific hub genes are found to be with potential brain development functions

The remarkable development of the brain in primate-lineage species, especially in human, is a decisive hallmark differentiating them from other organisms [28]. Recent studies have reported important roles of new genes in evolution of important human brain-related traits. For example, it was detected that an excess of young genes (that is, primate-specific) in the human genome are recruited in early human brain development [2]; potential strengthening functions of brain neoron-connection by SRGAP2 [29, 30]; the skin and brain functions by CHRFAM7A [31, 32]. We further investigated the correlation of the young genes in human that have evidence for functioning in brain development with their topological structures in the GGI networks.

**Fig. 7:**
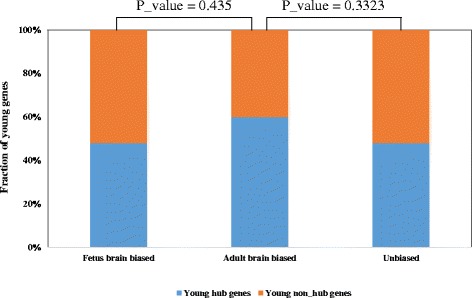
Comparison of PPI network topologies for young genes with diverse brain expression patterns. This figure shows the percentage distribution of young hub genes and young non-hub genes within different categories of brain expression patterns. The statistical significance difference was calculated using Fisher’s exact test

Through integrative analysis of the brain expression pattern data of these young genes [2] and their network topological features based on human PPI network data, we found no significant bias on the percentages of hub genes (with minimum interaction degrees of 6) among three different brain expression categories of young genes (Fisher’s exact test, Fetus vs. Adult: *P* value = 0.435, Adult vs. Unbiased: *P* value = 0.3323, Fig. 7). In other words, young genes with diverse network connectivity contribute equally during both early and late stages of human brain development.

**Fig. 8:**
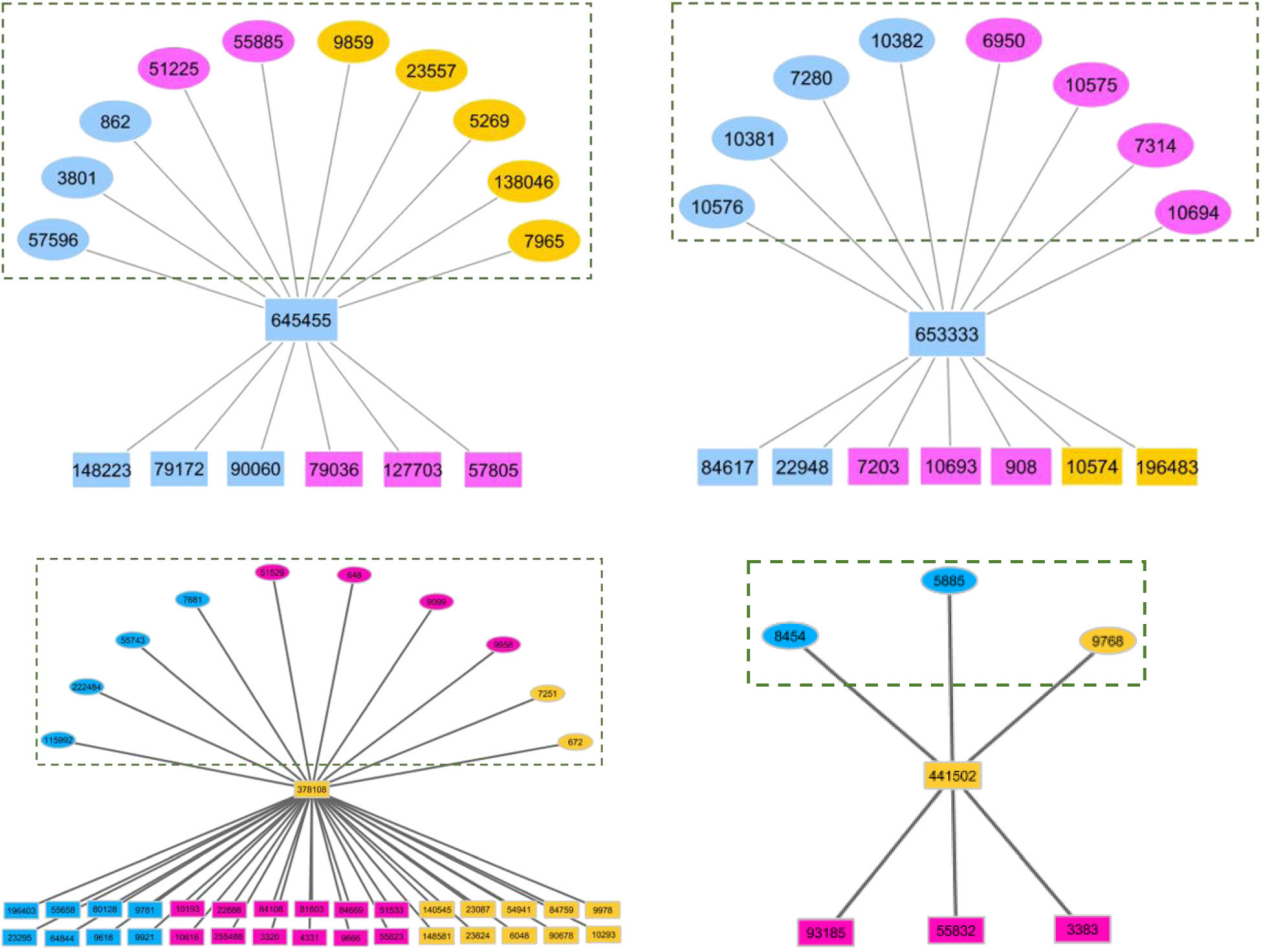
Human lineage-specific hub genes and their first-level linking partners. This figure illustrates two fetus brain biased human lineage-specific hub genes (top) and two unbiased human lineage-specific hub genes (bottom) and their direct interacting partners from the human PPI network. Genes biased in fetus brain (blue), adult brain (red), and unbiased (orange) between fetus and adult brain are marked. Genes (in square circles) outlined in the green dashed rectangle have been reported to have some brain development-related functions in previous literature

More intriguingly, four human-lineage specific (the genes that originated only in the human lineage since its divergence and thus exist only in the human genome) hub genes with clear expression evidence in human brain were found (Additional file 8: Table S4). As there was no direct clue in literatures about their functions in brain development of these four genes, we conducted a ‘guilt by connection’ study to investigate the reported evidence for the roles in brain function of their direct linking partners by manual curation of early studies (Additional file 9: Table S5). For instance, CCT4, a subunit of chaperonin containing TCP1, was reported to be involved with development of a brain malfunction disorder - Alzheimer’s disease [33], and it was also shown that CCT4 (gene id: 10575) is a direct interacting partner of one of young hub gene - FAM86B2 (gene id: 653333, Fig. 8). Collectively, we found that 62.5 % (10 of 16) and 53.3 % (8 of 15) of the first-layer linking partners for two out of the four hub genes, which were fetus brain biased, were confirmed to be involved in brain development (Fig. 8 and Additional file 9: Table S5). While for the other two unbiased hub genes, 24.4 % (10 out of 41) and 50 % (3 out of 6) were proven to function in brain development in previous literature (Fig. 8 and Additional file 9: Table S5). As genes with similar functions tend to be within the same network cluster [34], this evidence suggests these four human-lineage specific hub genes could also be with associated functions in human brain development.

### Multiple mechanisms drive the evolution of human GGI network

The most significant property of complex networks, including biological networks, is the power-law degree distribution [24] (Additional file 1: Figure S1), or so-called scale-free feature. Following the classic Barabasi-Albert (BA) model [35], this preferential attachment model was also applied to account for the scale-free feature of biological networks [36], which claims that new originating genes tend to interact with well-connected nodes. However, the biggest challenge for this model is the distinctive characteristics of biological networks - duplication as the dominant source of network evolution [37]. Therefore, another biologically motivated model called duplication-divergence model was proposed [38, 39], which accounts for both the gene duplication and the subsequent loss of inherited interactions. However, the acquirement of new links, except inherited interactions, was not considered in this model.

**Fig. 9:**
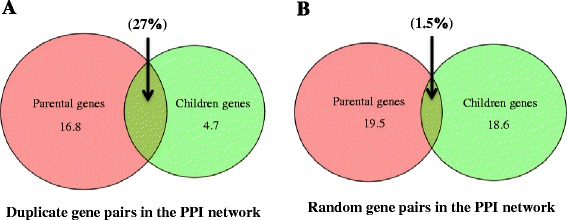
Inheritance of linking partners for duplication-based young genes (primate-specific genes). **a** The inheritance status for ‘real’ duplicate gene pairs in the context of PPI networks. **b** The inheritance status for random gene pairs in the context of PPI networks. The numbers inside the circles show the average PPI network connectivity for parental genes or children genes, and the percentages indicate the fractions of common linking partners shared by parental genes and children genes

**Fig. 10:**
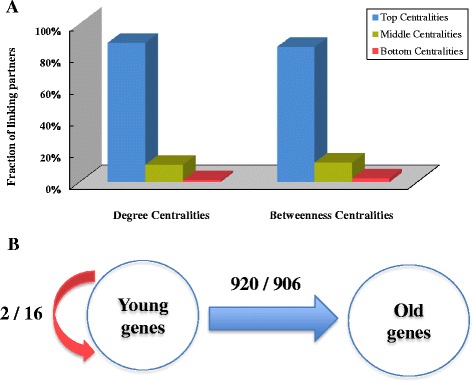
Preferential attachment to new linking partners of young genes (primate-specific genes) after removing the shared interactions with their parental genes. **a** Preference on network centralities (Degree and Betweenness) for young genes’ new interacting partners. The centralities are ranked and divided into three levels – high, moderate, and low, each with the same number of genes. **b** Young genes’ preference for the divergence times of new linking partners. The number before the slash line indicates the ‘real’ number of linking partners, while the number after is from random expectation

To address this issue from an evolutionary aspect, we defined primate-specific genes (branch 8–12 as shown in Fig. 1a) as young genes, and genes that originated before this time period as old genes. Among these young genes, 95 % of them were created from duplication-based (either from DNA-level duplication or RNA-level duplication) mechanisms (Additional file 10: Figure S5), which is in line with the classic argument that duplication is the dominant source of evolution [37]. Consequently, these young genes inherited on average 27 % linking partners from their parental genes (Fig. 9a), which is statistically greater (18 times) than that of random gene pairs (Fig. 9b). This finding indicated the inheritance of interacting partners of new genes from their parental copies [5]. We further explored the pattern for young genes to establish new linking partners, by removing those shared interactions with their parental genes. Different with the pattern in yeasts [10], we found that the young genes tend to prefer as new linking patterns the genes with high topological centralities (Chi-square tests, Degree: *P* value <2.2e-16; Betweenness: *P* value <2.2e-16, Fig. 10a) and elder age (Fisher’s exact test, *P* value = 0.001247, Fig. 10b), illuminating a rich-get-richer process [35] for new genes to develop new links. Thus, our results indicate the biological relevance of duplication-divergence model, and also show the preferential attachment to acquire novel links for new originating genes. This finding provided empirical data and new perspective for the development of new evolutionary models of biological networks in the future.

In this present study, we reported a gradual integration process of new genes into ancestral GGI networks (Fig. 2). An intriguing question to ask is what mechanisms are underlying the evolution of these new gene-integrated networks, or why new genes are generally less central in these GGI network. Based on these data, first, we proposed that the new genes-driven network evolution in humans is a mutation-limited process due to small effective population size [40]: as it is a time-dependent process for new genes to be adapted to the genome and GGI networks by establishing new linking partners.

In addition, new originating genes were found to be particularly shorter in protein length (Additional file 11: Figure S6A) [10], and consequently could only provide a limited interaction surface for potential interacting partners [41]. In the view of evolution, genes gradually evolve longer protein length to obtain more interactions, as they aged, indeed playing a role as one non-dominant mechanistic factor. However, we found that the shorter protein length was not a major factor to determine the links, as we observed the same patterns for the datasets of controlled protein lengths (Additional file 11: Figure S6B). Besides, new genes were also found to be expressed in fewer tissues (Fig. 5c and d) and lower expression levels (Additional file 11: Figure S6C), while genes with broader expression patterns (Fig. 5a and b) and higher expression levels (Additional file 11: Figure S6D) tend to have more interactions. Mechanically, the constraints on both the expression breadth (Fig. 5c and d) and expression levels (Additional file 11: Figure S6C) of new arising genes could only allow them to connect with genes expressed in the same tissues with limited binding space, which further hinder them from becoming highly connected nodes of the network. However, after being normalized by expression level and breadth, we found that given same expression levels and breadth the old genes still significantly evolved more links than young genes (Additional file 11: Figure S6E and F). Also, based on preceding analysis (Fig. 10), the highly connected older genes provide the new genes with more choices to develop new pathway(s) towards advantageous functions. Therefore, we concluded that, besides the mechanistic elements such as protein lengths and expression levels that may play a limited mechanistic role, the evolutionary time with the rich-get-richer preference of new linking partners have contributed significantly to the appearance of the observed evolution patterns of GGI networks that are impacted by evolutionary forces of natural selection and mutation.

Despite the general constraint on new genes to acquire linking partners (Fig. 2), we still found a fraction of new genes, especially young genes (primate-specific genes, branch 8–12, Fig. 1a), can rapidly evolve interactions and crush into network core (Fig. 4). It is tempting to ask what ‘fitness effect’ [42] facilitates the rapid acquirement of linking partners for these new genes. To address this issue, we explored the protein sequence features of those young hub genes (with minimum interaction degrees of 6) and young non-hub genes. Despite young hub genes being slightly shorter in protein length, they were found to be with larger proportions of low-complexity and intrinsic disordered regions than young non-hub genes (Additional file 12: Table S6). Low complexity and structural disorder regions create more flexibility and adaptability to bind distinct partners [41, 43]. Therefore, these beneficial intrinsic features endow these genes high-affinity to quickly acquire new interactions, therefore becoming network hubs.

## Conclusions

Our findings revealed a non-robust but rapid evolutionary process in which new genes are gradually integrated into ancestral GGI networks. We identified a few young genes that specifically exist in the human genome evolved into hubs in GGI networks, yielding important phenotypic effects in brain development.

## Methods

### Gene-gene interaction data

Human protein-protein interaction data were extracted and rescored from the 11 October 2013 release of interactions in the Database of Human Integrated Protein-Protein Interaction rEference (HIPPIE) [14], which integrated 18 public protein interaction data sources. Each interaction was assigned a confidence score according to the number and the quality of experimental techniques utilized for the detection of this interaction, and interlog cases in other model organisms. To avoid missing species-specific interactions, the filtering parameter of interlogs in model organisms was omitted. A medium confidence level (0.68 - the median of score distribution) was set as the threshold, and interactions with confidence scores no smaller than this cutoff were retrieved. Self-interactions were excluded in this study. To eliminate the bias from arbitrary choice of cutoff, another human PPI network was reconstructed with a stricter threshold of confidence score (0.77). Hub nodes are defined as genes with minimum interaction degree of 6, which is the medium level connectivity of global human PPI network. To further avoid the potential bias from data collection, we also utilized another manual curated human PPI dataset – Human Protein Reference Database (HPRD release version 9) [17]. Similarly, self-interactions were eliminated from this dataset, and only non-redundant interactions were retained.

Mouse protein-protein interaction data was integrated from five well-collected datasets (Additional file 5: Table S2). The confidence score assignment of each interaction followed that of HIPPIE [14], except the removal of the filtering parameter of interlogs as aforementioned. The self-interactions were also excluded from the dataset. Similarly, a moderate confidence score (0.68 - the median of score distribution) was set as the threshold to define reliable interaction pairs. Herein, proteins were considered to be equivalent to their protein-coding genes, and assigned with the same gene identifiers through a web ID conversion tool – bioDBnet [44].

Based on gene expression profiling data of 65 human tissues collected from a public co-expressed gene database (COXPRESdb v5.0) [18], we constructed a human gene co-expression (GC) network by exploring the expression profile associations between pair-wise genes, indicated by Pearson correlation coefficients (PCC) [45]. To get a human GC network with comparable number of gene nodes to be human PPI network (Additional file 2: Table S1) and biologically relevant (Additional file 3: Figure S2), gene pairs were considered linked if their expression association with PCC was greater than 0.4.

### Gene age and origination mechanism data

Both human and mouse gene age data were retrieved from an early study by Yong *et al.* [13]. In brief, each protein-coding gene was dated and given branch assignment by inferring the absence and presence of orthologs along the vertebrate phylogenetic tree (Fig. 1a and Additional file 6: Figure S4A), based on UCSC syntenic genomic alignment. This gene dating strategy was reported to be conservative and sensitive for identification of fast-evolving genes [2]. The origination mechanism information of human young genes (primate-specific genes) was from the same study. Young genes that originated from DNA-level duplication or RNA-level duplication were annotated as duplication-originating genes, otherwise were defined as *de novo* genes. Additionally, we also used another human gene origin data based on phylostratigraphic analysis [19], which assigned human genes with phylogenetic branches from 1 to 19, based on the absence and presence of orthologs in the genomes through cellular organisms to primate species (Additional file 4: Figure S3A). The detailed information about all these gene age datasets can be found in Additional file 13: Table S7.

### Human gene expression profiling data

The mRNA and protein expression profiling data for human tissues were extracted from the Human Protein Atlas Project (V12) [20], which was launched for systematic exploration of the human proteome. RNA-seq technique was exploited to probe the mRNA expression patterns of 20,315 human genes in 27 tissues, and genes with FPKM (fragments per kilobase of exon per million reads mapped) greater than 1.0 were defined as expressed within specific tissues. Antibody-based proteomics were used for profiling the expression of proteins for 16,384 human coding genes in 58 tissues, and only proteins with clear bands detected from western blots within corresponding tissues were defined as expression.

### Human essential genes information

Essential genes are defined as those genes that are critical for the survival of an organism. In this study, potential gene essential information were collected from four distinct resources – (1) genes associating with the most life-threatening diseases, which can cause death prior to puberty, or infertility of individuals [46]; (2) combinational essential genes detected from large-scale human diseases cell lines via RNA inference (RNAi) experiments [47] and a recently emerging technology called CRISPR-Cas9 system [48]; (3) functional essential genes collected from independent studies via text-mining methods [49]; and (4) orthologous genes of genes that are essential in mouse, detected by gene knock-out experiments [50]. Finally, 1,342 genes that co-exist within two or more above datasets were defined as human essential genes (Additional file 7: Table S3).

### Calculation for network topological features

Two topological centrality parameters, that is, Degree (or connectivity), Betweenness, were used to measure genes’ centralities in the GGI networks. Degree centrality is a basic property, which indicates the number of adjacent edges a node bears. Betweenness is an index to the measure the importance of one vertex to the shortest paths among other nodes in the network [51]:

$$ \mathrm{B}\left(\mathrm{v}\right)={\displaystyle \sum_{i\ne j\ne v\notin V}\frac{\mathrm{K}\left(\mathrm{i}\mathrm{v}\mathrm{j}\right)}{\mathrm{K}\left(\mathrm{i}\mathrm{j}\right)}} $$

**K(ij):** The number of shortest paths between vertex i and vertex j.

**K(ivj):** The number of shortest paths between vertex i and vertex j, which go through vertex v.

All of these calculations were implemented on R platform [52], by exploiting a network analysis R package referred to as igraph [53]. The visualization of sub-networks in this study was conducted with a widely exploited software - Cytoscape [54].

### Sequence feature analysis for human proteins

Three intrinsic features of protein sequences were calculated – protein length, low complexity region, and structural disorder region. Human protein sequences were downloaded from Ensembl database [55]. If one gene has alternative splicing isoforms, the protein with longest length was retrieved from further analysis. The existing program SEG was used to detect the low-complexity regions in protein sequences [56], by default parameter setup. As the experimentally validated information for disorder proteins was in deficiency [57], the disorder regions of protein sequences were predicted via an online predictor – IUPred [58, 59]. One residue was defined as intrinsic disorder, if the calculation score was greater than 0.5 [58]. Two modes (long disorder and short disorder, respectively) of this program were separately applied for the prediction of structural disorder regions.

### Data availability

The detailed information and download links for all the datasets used in this study can be accessed via http://longlab.uchicago.edu/?q=SD_GB.

## Additional files

Additional file 1: Figure S1. Power-law degree distributions of Human PPI networks reconstructed from HIPPIE with confidence score threshold of 0.68 (A) and 0.77 (B). (PDF 111 kb) Additional file 2: Table S1. General characteristics of GGI networks. (PDF 91 kb) Additional file 3: Figure S2. Characteristics of other human GGI networks. (PDF 116 kb) Additional file 4: Figure S3. PPI network topological patterns of human genes in relation to their phylogenetic groups from another gene age dataset. (PDF 85 kb) Additional file 5: Table S2. Summary of Mouse PPI datasets integrated in this study. (PDF 7 kb) Additional file 6: Figure S4. PPI network topological patterns of mouse genes in relation to divergence times. (PDF 146 kb) Additional file 7: Table S3. Summary of human gene essentiality data used in this study. (PDF 1107 kb) Additional file 8: Table S4. Characteristics of network topology and brain expression pattern for human lineage-specific hub genes. (PDF 7 kb) Additional file 9: Table S5. Summary of brain-function related genes with literature evidence. (PDF 97 kb) Additional file 10: Figure S5. Distribution of young genes (primate-specific genes) that originated from duplication-based and *de novo* mechanisms. (PDF 48 kb) Additional file 11: Figure S6. Comparison of gene features between young genes (primate-specific) and old genes. (PDF 114 kb) Additional file 12: Table S6. Protein sequence features of young hubs and young non-hubs. (PDF 10 kb) Additional file 13: Table S7. Gene age datasets used in this study. (XLS 5395 kb)

## Abbreviations

GC: Gene co-expression
GGI: Gene-gene interaction
hGC: Human gene co-expression
hPPI: Human protein-protein interactions
mPPI: Mouse protein-protein interactions
PPI: Protein-protein interaction
