# Supplementary material for: New genes drive the evolution of gene interaction networks in the human and mouse genomes
Source: Genome Biol. 2015 Oct 1;16:202. doi: 10.1186/s13059-015-0772-4 (PMC4590697; doi:10.1186/s13059-015-0772-4)
Supplement: Additional file 1: Figure S1. — Power-law degree distributions of Human PPI networks reconstructed from HIPPIE with confidence score threshold of 0.68 (A) and 0.77 (B). (PDF 111 kb) [file 13059_2015_772_MOESM1_ESM.pdf]

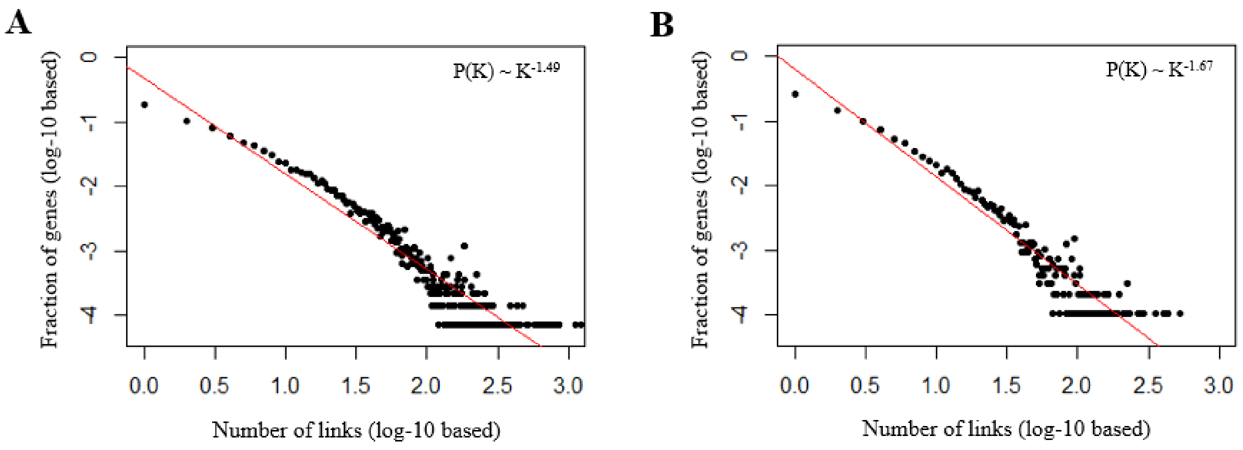

Figure S1 Power-law degree distributions of Human PPI networks reconstructed from HIPPIE with confidence score threshold of 0.68 (A) and 0.77 (B). The x-axis indicates the degrees (numbers of interactions) of genes in log10-based transformation, and the y-axis shows the percentage of genes (log10-based) with the corresponding degree. The red lines exhibit the line of the best fit for these distributions, with degree exponents of 1.49 and 1.67, respectively.
