# Supplementary material for: New genes drive the evolution of gene interaction networks in the human and mouse genomes
Source: Genome Biol. 2015 Oct 1;16:202. doi: 10.1186/s13059-015-0772-4 (PMC4590697; doi:10.1186/s13059-015-0772-4)
Supplement: Additional file 2: Table S1. — General characteristics of GGI networks. (PDF 91 kb) [file 13059_2015_772_MOESM2_ESM.pdf]

Table S1: General characteristics of GGI networks

Organism	Network type	#Nodes	#Interactions	<K>	Υ	C	C_{rand}	ι	ι_{rand}
Human	**PPI network** (HIPPIE, [1]Cutoff: 0.68)	13987	130761*2	18.7	1.50	0.051	0.0013	3.34	3.60
	**PPI network** (HIPPIE, [1]Cutoff: 0.77)	9656	45842*2	9.5	1.67	0.071	0.0012	3.97	4.33
	**PPI network** (HPRD)	8621	32342*2	7.5	1.81	0.055	0.0008	4.26	4.7
	**GC network** ([2]Cutoff: 0.4)	13123	794265*2	121	1.19	0.457	0.0092	3.23	2.32
Mouse	**PPI network** ([1]Cutoff: 0.68)	4633	9921*2	4.3	1.49	0.029	0.0010	4.36	6.04

Abbreviations:
PPI: Protein-Protein Interaction; GC: Gene Co-expression;
[1]Confidence score; [2]Pearson correlation coefficient;
#Nodes: Number of nodes; #Interactions: Number of interacting links; <K>: Average connectivity (degree) of network;
Υ: Degree exponent of scale-free network; C: Clustering coefficient of network; ι: Average path length of network;
C_{rand}, ι_{rand}: Clustering coefficient and average path length of random network with the same numbers of nodes and interactions.
