# Supplementary material for: New genes drive the evolution of gene interaction networks in the human and mouse genomes
Source: Genome Biol. 2015 Oct 1;16:202. doi: 10.1186/s13059-015-0772-4 (PMC4590697; doi:10.1186/s13059-015-0772-4)
Supplement: Additional file 3: Figure S2. — Characteristics of other human GGI networks. (PDF 116 kb) [file 13059_2015_772_MOESM3_ESM.pdf]

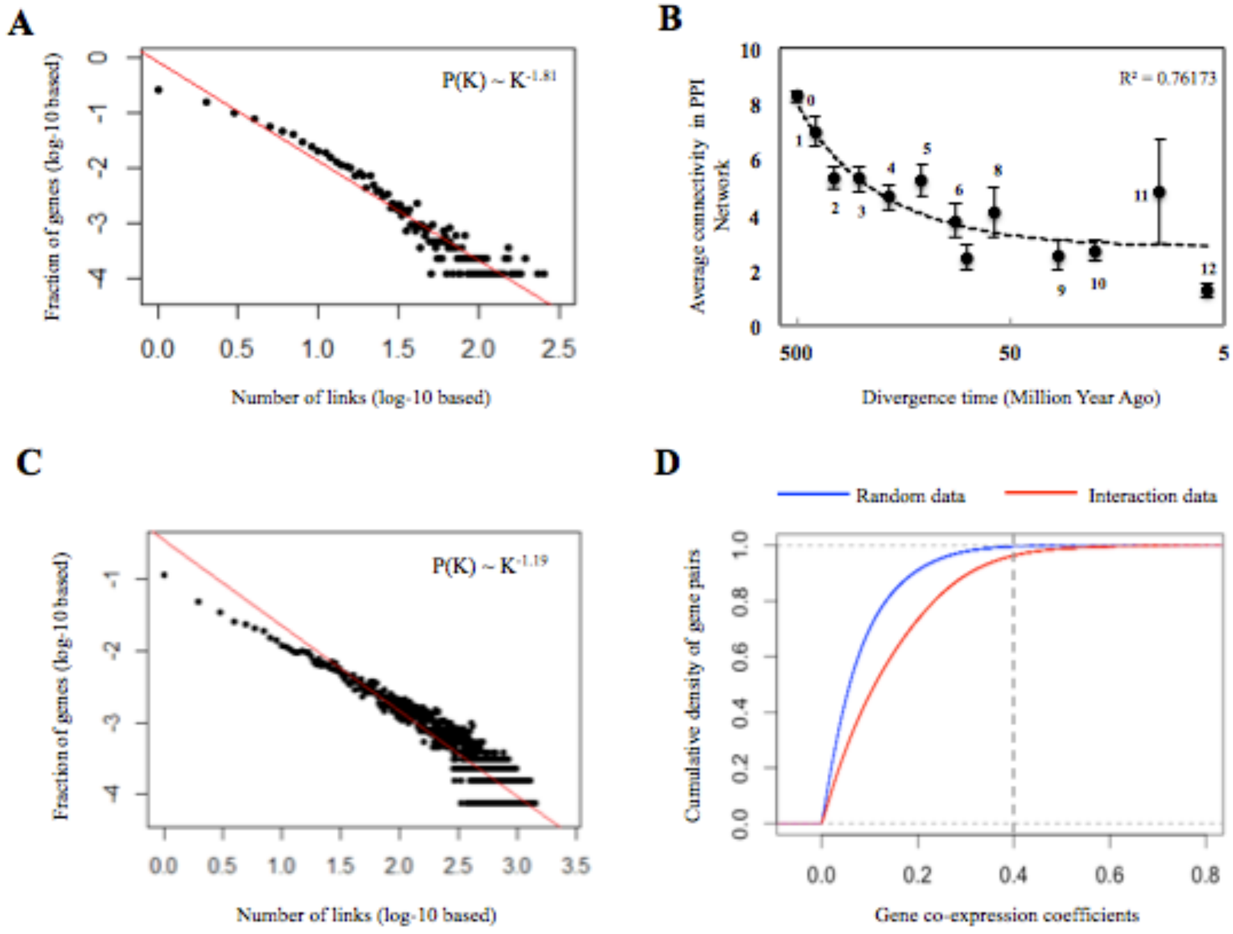

Figure S2 Characteristics of other human GGI networks. (A) Power-law degree distribution of the PPI network based on HPRD. **(B)** Distribution of PPI network connectivity (numbers of interactions) for genes from different phylogenetic branches. The error bars show the standard error of the mean for each group of genes, and the dash line indicates the polynomial regression correlation between network connectivity of genes and their divergence times. Numbers near each data point are phylogenetic branch assignments for each group of genes. The divergence time of each gene age group was assigned as the middle time point for each branch. And the oldest branch (branch 0) is arbitrarily set as 500 myr. (**C)** Power-law degree distribution of the human GC network. (**D**) The cumulative density distribution in relation to gene co-expression (Pearson's correlation) coefficients of gene pairs with physical protein-protein interactions (red) and from random selection (blue). The grey dashed line indicates the cut-off of gene co-expression coefficient (0.4) for the construction of human GC network in this present work. For (A) and (C), the x-axis indicates the degrees (numbers of interactions) of genes in log10-based transformation, and the y-axis shows the percentage of genes (log10-based) with the corresponding degree. The red line exhibits the line of the best fit for this distribution, with a degree exponent of 1.81 and 1.19, respectively.
