# Supplementary material for: New genes drive the evolution of gene interaction networks in the human and mouse genomes
Source: Genome Biol. 2015 Oct 1;16:202. doi: 10.1186/s13059-015-0772-4 (PMC4590697; doi:10.1186/s13059-015-0772-4)
Supplement: Additional file 4: Figure S3. — PPI network topological patterns of human genes in relation to their phylogenetic groups from another gene age dataset. (PDF 85 kb) [file 13059_2015_772_MOESM4_ESM.pdf]

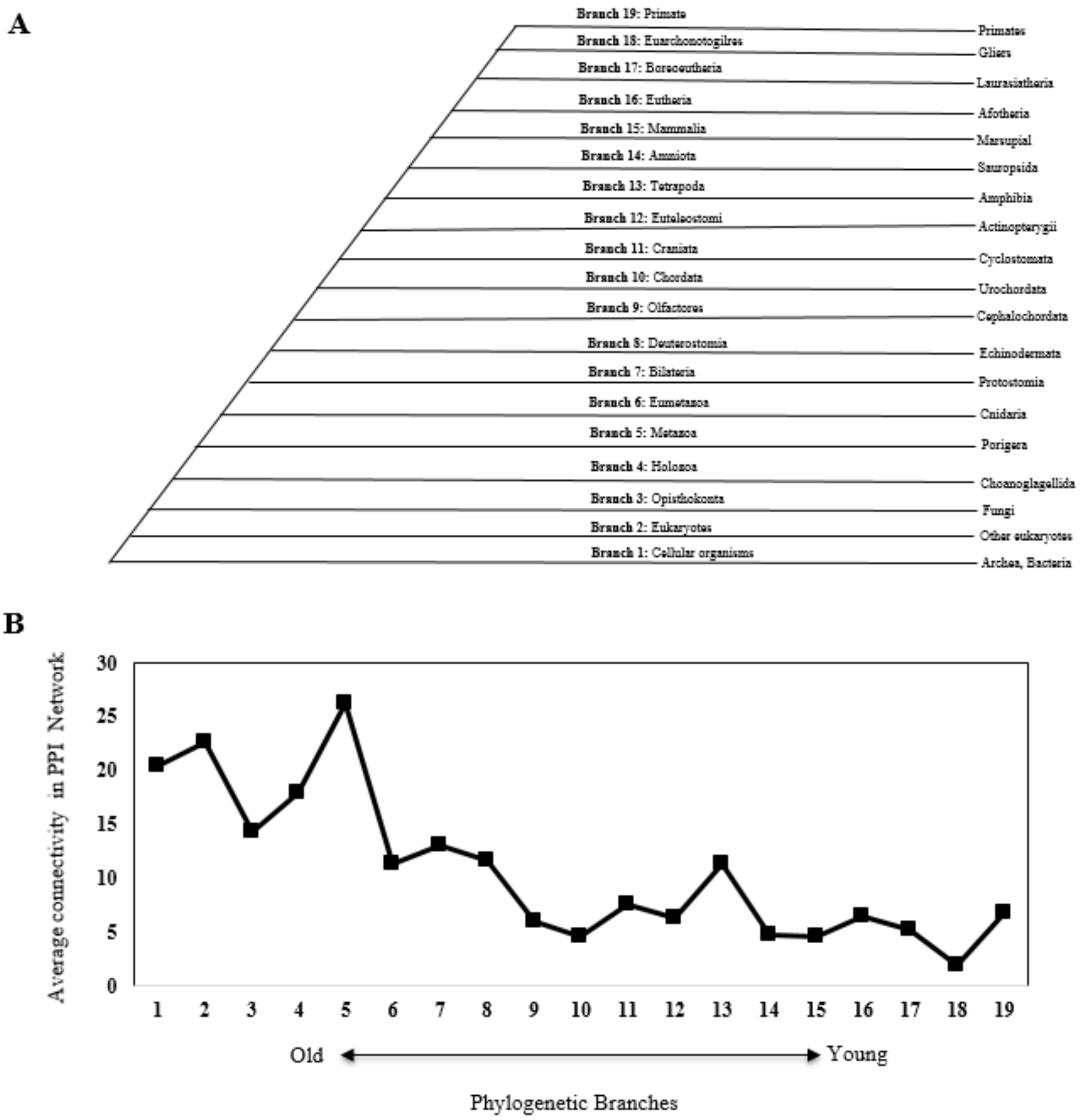

Figure S3 PPI network topological patterns of human genes in relation to their phylogenetic groups. **(A)** Phylogenetic tree of life towards human from another dataset. **(B)** Distribution of PPI network connectivity (numbers of interactions) for genes from different phylogenetic branches.
