# Supplementary material for: New genes drive the evolution of gene interaction networks in the human and mouse genomes
Source: Genome Biol. 2015 Oct 1;16:202. doi: 10.1186/s13059-015-0772-4 (PMC4590697; doi:10.1186/s13059-015-0772-4)
Supplement: Additional file 5: Table S2. — Summary of Mouse PPI datasets integrated in this study. (PDF 7 kb) [file 13059_2015_772_MOESM5_ESM.pdf]

Table S2: Summary of Mouse PPI datasets integrated in this study

PPI dataset	Version	Data Size *	Reference (PMID)
BioGrid	V-3.1.94	7372	16381927
DIP	2012.08.18	414	10592249
Intact	2012.10.04	8933	14681455
MINT	2012-10-29	2425	17135203
MPPI	2012-03-02	464	15531608
**Total**		19608	

***Data Size:** Number of interactions within the dataset.
