# Supplementary material for: New genes drive the evolution of gene interaction networks in the human and mouse genomes
Source: Genome Biol. 2015 Oct 1;16:202. doi: 10.1186/s13059-015-0772-4 (PMC4590697; doi:10.1186/s13059-015-0772-4)
Supplement: Additional file 6: Figure S4. — PPI network topological patterns of mouse genes in relation to divergence times. (PDF 146 kb) [file 13059_2015_772_MOESM6_ESM.pdf]

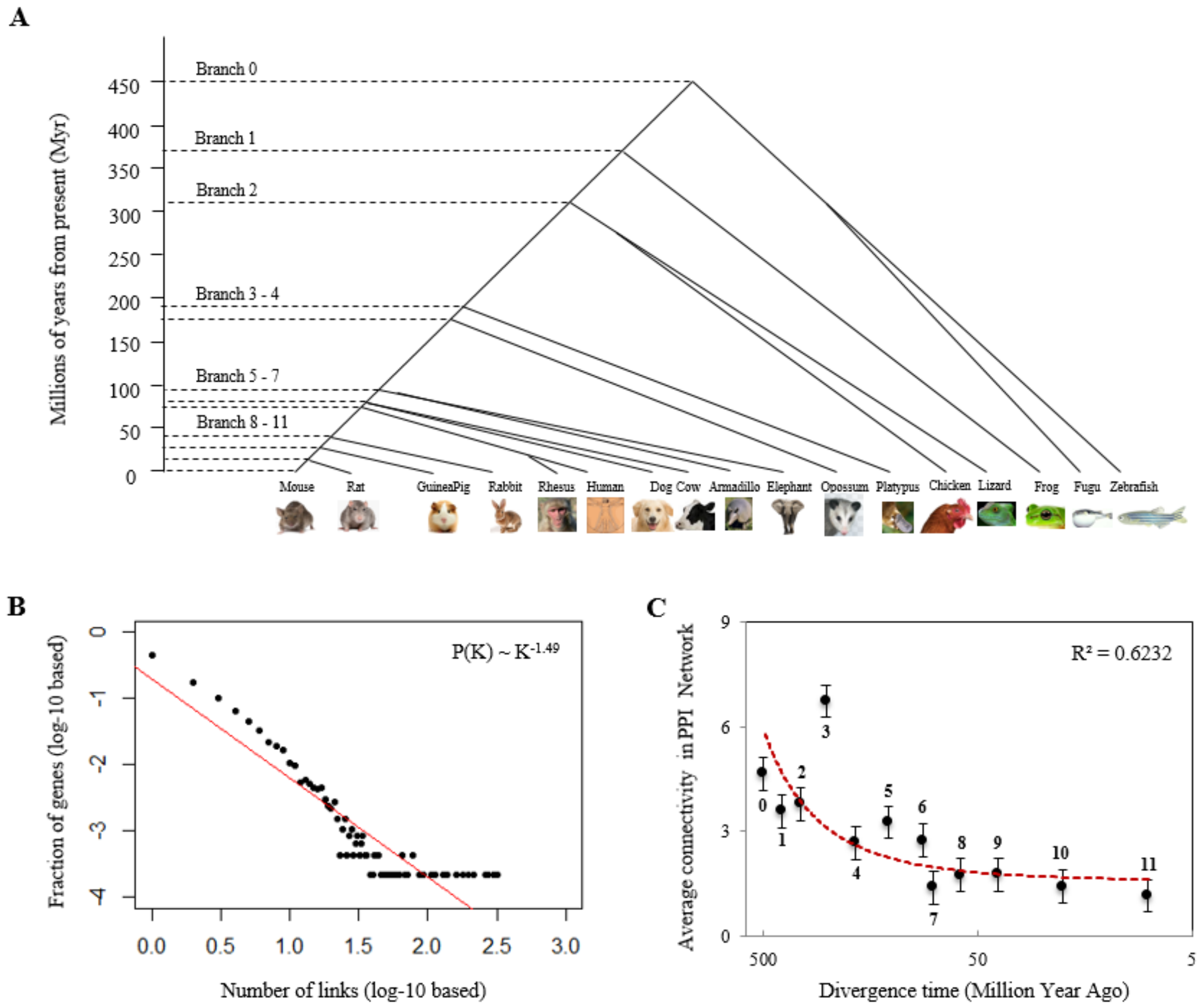

Figure S4 PPI network topological patterns of mouse genes in relation to divergence times. (A) Phylogenetic tree of vertebrates towards mouse with branches and divergence time in myr from present. **(B)** Power-law degree distribution of mouse PPI network. The x-axis indicates the degrees (numbers of interactions) of genes in log10-based transformation, and the y-axis shows the percentage of genes (log10-based) with the corresponding degree. The red line exhibits the line of the best fit for this distribution, with a degree exponent of 1.49. **(C)** Distribution of PPI network connectivity (numbers of interactions) for genes from different phylogenetic branches. The error bars show the standard error of the mean for each group of genes, and the dash line indicates the polynomial regression correlation between network connectivity of genes and their divergence times. Numbers near each data point are phylogenetic branch assignments for each group of genes. The divergence time of each gene age group was assigned as the middle time point for each branch. And the oldest branch (branch 0) is arbitrarily set as 500 myr.
