# Supplementary material for: New genes drive the evolution of gene interaction networks in the human and mouse genomes
Source: Genome Biol. 2015 Oct 1;16:202. doi: 10.1186/s13059-015-0772-4 (PMC4590697; doi:10.1186/s13059-015-0772-4)
Supplement: Additional file 7: Table S3. — Summary of human gene essentiality data used in this study. (PDF 1107 kb) [file 13059_2015_772_MOESM7_ESM.pdf]

Table S3: Summary of human gene essentiality data used in this study.

Phylogenetic branch	Ensembl ID	Associating disease and OMIM ID	Cause Death prior to puberty	Cause Infertility	Evidence from cell line experiments	Evidence from text mining	Evidence from mouse essential orthologs
11	ENSG00000126524	-	-	-	YES	NO	YES
9	ENSG00000198670	-	-	-	YES	YES	NO
5	ENSG00000095752	-	-	-	YES	YES	NO
5	ENSG00000105568	-	-	-	YES	NO	YES
5	ENSG00000107262	-	-	-	NO	YES	YES
5	ENSG00000135446	-	-	-	YES	NO	YES
5	ENSG00000143546	-	-	-	YES	NO	YES
5	ENSG00000164399	-	-	-	YES	YES	NO
5	ENSG00000164400	-	-	-	YES	YES	YES
5	ENSG00000177143	-	-	-	YES	YES	NO
5	ENSG00000179142	Corticosterone methyloxidase type II deficiency, 610600	YES	NO	NO	NO	YES
5	ENSG00000182636	-	-	-	YES	NO	YES
5	ENSG00000183734	-	-	-	NO	YES	YES
5	ENSG00000185479	-	-	-	YES	NO	YES
4	ENSG00000104432	-	-	-	YES	YES	NO
4	ENSG00000108518	-	-	-	YES	NO	YES
4	ENSG00000115297	-	-	-	NO	YES	YES
4	ENSG00000125538	-	-	-	YES	YES	NO
4	ENSG00000131462	-	-	-	YES	NO	YES
4	ENSG00000162244	-	-	-	YES	NO	YES
4	ENSG00000163554	-	-	-	NO	YES	YES
4	ENSG00000164885	-	-	-	YES	YES	YES
4	ENSG00000173805	-	-	-	NO	YES	YES
4	ENSG00000184500	-	-	-	NO	YES	YES
4	ENSG00000204256	-	-	-	YES	NO	YES
3	ENSG00000002330	-	-	-	YES	YES	NO
3	ENSG00000028137	-	-	-	YES	YES	NO
3	ENSG00000049540	-	-	-	NO	YES	YES
3	ENSG00000055332	-	-	-	YES	YES	NO
3	ENSG00000070814	-	-	-	YES	NO	YES
3	ENSG00000102265	-	-	-	YES	YES	NO
3	ENSG00000104164	-	-	-	YES	YES	NO
3	ENSG00000106331	-	-	-	YES	NO	YES
3	ENSG00000111321	-	-	-	YES	YES	NO
3	ENSG00000113520	-	-	-	YES	YES	NO
3	ENSG00000115008	-	-	-	YES	YES	NO
3	ENSG00000117525	-	-	-	YES	YES	YES
3	ENSG00000125726	-	-	-	YES	YES	NO
3	ENSG00000130427	-	-	-	NO	YES	YES
3	ENSG00000132535	-	-	-	YES	NO	YES
3	ENSG00000133710	-	-	-	NO	YES	YES

3	ENSG00000134460	-	-	-	YES	YES	NO
3	ENSG00000136244	-	-	-	YES	YES	NO
3	ENSG00000139193	-	-	-	YES	YES	NO
3	ENSG00000145839	-	-	-	YES	YES	NO
3	ENSG00000164825	-	-	-	YES	NO	YES
3	ENSG00000173327	-	-	-	NO	YES	YES
3	ENSG00000196154	-	-	-	YES	NO	YES
2	ENSG00000003436	-	-	-	NO	YES	YES
2	ENSG00000006634	-	-	-	YES	YES	NO
2	ENSG00000012048	-	-	-	YES	YES	YES
2	ENSG00000014257	-	-	-	YES	YES	NO
2	ENSG00000026508	-	-	-	YES	YES	NO
2	ENSG00000030110	-	-	-	YES	YES	NO
2	ENSG00000049130	-	-	-	NO	YES	YES
2	ENSG00000073009	-	-	-	NO	YES	YES
2	ENSG00000085662	-	-	-	YES	YES	NO
2	ENSG00000089289	-	-	-	YES	YES	NO
2	ENSG00000091513	-	-	-	YES	YES	YES
2	ENSG00000092330	-	-	-	YES	NO	YES
2	ENSG00000099810	-	-	-	NO	YES	YES
2	ENSG00000100387	-	-	-	YES	NO	YES
2	ENSG00000106991	-	-	-	YES	YES	YES
2	ENSG00000107562	-	-	-	NO	YES	YES
2	ENSG00000108064	-	-	-	YES	NO	YES
2	ENSG00000114251	-	-	-	YES	NO	YES
2	ENSG00000115163	-	-	-	YES	NO	YES
2	ENSG00000117400	-	-	-	YES	YES	NO
2	ENSG00000128342	-	-	-	YES	YES	YES
2	ENSG00000132604	-	-	-	YES	YES	YES
2	ENSG00000132676	-	-	-	YES	NO	YES
2	ENSG00000134086	-	-	-	YES	NO	YES
2	ENSG00000134531	-	-	-	YES	YES	NO
2	ENSG00000134690	-	-	-	YES	NO	YES
2	ENSG00000135744	-	-	-	NO	YES	YES
2	ENSG00000136634	-	-	-	YES	YES	NO
2	ENSG00000137812	-	-	-	YES	NO	YES
2	ENSG00000145241	-	-	-	YES	NO	YES
2	ENSG00000147889	-	-	-	YES	YES	NO
2	ENSG00000162692	-	-	-	YES	YES	YES
2	ENSG00000164045	-	-	-	YES	YES	YES
2	ENSG00000166548	-	-	-	YES	NO	YES
2	ENSG00000167642	-	-	-	NO	YES	YES
2	ENSG00000168040	-	-	-	YES	YES	YES
2	ENSG00000168878	-	-	-	NO	YES	YES
2	ENSG00000171867	-	-	-	YES	YES	NO
2	ENSG00000172613	-	-	-	YES	NO	YES

2	ENSG00000174697	Obesity, severe, due to leptin deficiency; Obesity, morbid, with hypogonadism, 601665	NO	YES	NO	YES	NO
2	ENSG00000175793	-	-	-	YES	NO	YES
2	ENSG00000178562	-	-	-	YES	YES	NO
2	ENSG00000184371	-	-	-	YES	NO	YES
2	ENSG00000187266	-	-	-	YES	YES	YES
2	ENSG00000187608	-	-	-	YES	YES	NO
2	ENSG00000188690	-	-	-	NO	YES	YES
2	ENSG00000197905	-	-	-	NO	YES	YES
2	ENSG00000198125	-	-	-	NO	YES	YES
1	ENSG00000018236	-	-	-	YES	YES	YES
1	ENSG00000049768	-	-	-	NO	YES	YES
1	ENSG00000067225	-	-	-	YES	NO	YES
1	ENSG00000078246	-	-	-	YES	NO	YES
1	ENSG00000078401	-	-	-	NO	YES	YES
1	ENSG00000090932	Spondylocostal dysostosis, autosomal recessive, 1, 277300	YES	NO	YES	NO	YES
1	ENSG00000100311	-	-	-	YES	NO	YES
1	ENSG00000102678	-	-	-	YES	NO	YES
1	ENSG00000103241	-	-	-	NO	YES	YES
1	ENSG00000104320	-	-	-	YES	YES	YES
1	ENSG00000105610	-	-	-	YES	YES	YES
1	ENSG00000106089	-	-	-	NO	YES	YES
1	ENSG00000111319	-	-	-	NO	YES	YES
1	ENSG00000113594	-	-	-	NO	YES	YES
1	ENSG00000114166	-	-	-	YES	YES	NO
1	ENSG00000114200	-	-	-	YES	YES	NO
1	ENSG00000115310	-	-	-	NO	YES	YES
1	ENSG00000117560	-	-	-	YES	YES	NO
1	ENSG00000119888	-	-	-	YES	NO	YES
1	ENSG00000128714	-	-	-	NO	YES	YES
1	ENSG00000129991	-	-	-	NO	YES	YES
1	ENSG00000131477	-	-	-	YES	NO	YES
1	ENSG00000131747	-	-	-	YES	YES	NO
1	ENSG00000135374	-	-	-	YES	NO	YES
1	ENSG00000137270	-	-	-	NO	YES	YES
1	ENSG00000138279	-	-	-	NO	YES	YES
1	ENSG00000140650	-	-	-	YES	YES	YES
1	ENSG00000145675	-	-	-	YES	NO	YES
1	ENSG00000148908	-	-	-	YES	NO	YES
1	ENSG00000152422	-	-	-	YES	YES	YES
1	ENSG00000153094	-	-	-	NO	YES	YES
1	ENSG00000159399	-	-	-	YES	NO	YES
1	ENSG00000164867	-	-	-	NO	YES	YES
1	ENSG00000168447	Autosomal recessive pseudohypoaldosteronism type I, 264350	YES	NO	NO	NO	YES

1	ENSG00000170348	-	-	-	YES	NO	YES
1	ENSG00000172115	-	-	-	YES	NO	YES
1	ENSG00000172179	-	-	-	YES	YES	NO
1	ENSG00000176692	-	-	-	YES	YES	YES
1	ENSG00000179776	-	-	-	NO	YES	YES
1	ENSG00000184047	-	-	-	YES	YES	NO
1	ENSG00000184408	-	-	-	YES	YES	NO
1	ENSG00000186395	-	-	-	YES	NO	YES
1	ENSG00000189403	-	-	-	NO	YES	YES
1	ENSG00000196811	Lethal multiple pterygium syndrome, 253290	YES	NO	NO	YES	YES
1	ENSG00000196924	-	-	-	NO	YES	YES
1	ENSG00000206075	-	-	-	YES	YES	YES
0	ENSG00000001084	-	-	-	NO	YES	YES
0	ENSG00000001626	-	-	-	NO	YES	YES
0	ENSG00000001630	-	-	-	NO	YES	YES
0	ENSG00000001631	-	-	-	YES	YES	YES
0	ENSG00000002822	-	-	-	YES	NO	YES
0	ENSG00000003056	-	-	-	YES	NO	YES
0	ENSG00000004478	-	-	-	YES	NO	YES
0	ENSG00000004487	-	-	-	YES	NO	YES
0	ENSG00000004848	-	-	-	NO	YES	YES
0	ENSG00000004939	-	-	-	YES	NO	YES
0	ENSG00000004975	-	-	-	YES	NO	YES
0	ENSG00000005884	-	-	-	YES	NO	YES
0	ENSG00000007168	Lissencephaly-1 (subcortical laminar heterotopia, included), 607432; Miller-Dieker lissencephaly syndrome, 247200	YES	NO	NO	YES	YES
0	ENSG00000007237	-	-	-	YES	YES	NO
0	ENSG00000007314	-	-	-	NO	YES	YES
0	ENSG00000007372	-	-	-	NO	YES	YES
0	ENSG00000008441	-	-	-	NO	YES	YES
0	ENSG00000008710	-	-	-	YES	YES	YES
0	ENSG00000008988	-	-	-	YES	NO	YES
0	ENSG00000009413	-	-	-	YES	YES	YES
0	ENSG00000010017	-	-	-	YES	NO	YES
0	ENSG00000010671	-	-	-	YES	YES	NO
0	ENSG00000010810	-	-	-	YES	YES	NO
0	ENSG00000011143	-	-	-	NO	YES	YES
0	ENSG00000011198	-	-	-	NO	YES	YES
0	ENSG00000011465	-	-	-	YES	YES	NO
0	ENSG00000012983	-	-	-	YES	YES	NO
0	ENSG00000013275	-	-	-	YES	NO	YES
0	ENSG00000013375	-	-	-	NO	YES	YES
0	ENSG00000017260	-	-	-	NO	YES	YES
0	ENSG00000017427	-	-	-	YES	YES	YES
0	ENSG00000018408	-	-	-	YES	NO	YES

0	ENSG00000018510	-	-	-	YES	NO	YES
0	ENSG00000019991	-	-	-	YES	YES	YES
0	ENSG00000020181	-	-	-	YES	NO	YES
0	ENSG00000020426	-	-	-	YES	NO	YES
0	ENSG00000020922	-	-	-	NO	YES	YES
0	ENSG00000021826	Carbamoylphosphate synthetase I deficiency, 237300; Familial persistent pulmonary hypertension of the newborn, 265380	YES	NO	NO	NO	YES
0	ENSG00000023734	-	-	-	YES	NO	YES
0	ENSG00000025156	-	-	-	NO	YES	YES
0	ENSG00000026025	-	-	-	YES	YES	NO
0	ENSG00000026036	-	-	-	NO	YES	YES
0	ENSG00000027075	-	-	-	YES	NO	YES
0	ENSG00000028203	-	-	-	NO	YES	YES
0	ENSG00000029639	-	-	-	NO	YES	YES
0	ENSG00000030304	-	-	-	YES	YES	YES
0	ENSG00000032444	-	-	-	YES	YES	YES
0	ENSG00000034063	-	-	-	YES	NO	YES
0	ENSG00000035403	-	-	-	NO	YES	YES
0	ENSG00000036257	-	-	-	YES	NO	YES
0	ENSG00000036473	-	-	-	NO	YES	YES
0	ENSG00000038382	-	-	-	YES	NO	YES
0	ENSG00000038427	-	-	-	NO	YES	YES
0	ENSG00000039068	-	-	-	YES	YES	YES
0	ENSG00000041982	-	-	-	YES	YES	NO
0	ENSG00000043355	Holoprosencephaly-5, 609637	YES	NO	NO	NO	YES
0	ENSG00000043462	-	-	-	YES	NO	YES
0	ENSG00000044524	-	-	-	YES	NO	YES
0	ENSG00000044574	-	-	-	NO	YES	YES
0	ENSG00000046604	-	-	-	NO	YES	YES
0	ENSG00000046651	-	-	-	YES	YES	NO
0	ENSG00000048052	-	-	-	YES	YES	NO
0	ENSG00000048707	-	-	-	YES	NO	YES
0	ENSG00000050748	-	-	-	YES	YES	NO
0	ENSG00000050820	-	-	-	YES	NO	YES
0	ENSG00000051180	-	-	-	YES	YES	YES
0	ENSG00000051382	-	-	-	YES	NO	YES
0	ENSG00000053747	-	-	-	NO	YES	YES
0	ENSG00000055118	-	-	-	NO	YES	YES
0	ENSG00000055130	-	-	-	YES	YES	YES
0	ENSG00000055208	-	-	-	NO	YES	YES
0	ENSG00000057593	-	-	-	NO	YES	YES
0	ENSG00000057663	-	-	-	YES	NO	YES
0	ENSG00000058085	-	-	-	NO	YES	YES
0	ENSG00000058272	-	-	-	YES	NO	YES
0	ENSG00000060069	-	-	-	YES	YES	NO

0	ENSG00000060237	-	-	-	YES	NO	YES
0	ENSG00000060718	-	-	-	YES	NO	YES
0	ENSG00000064012	-	-	-	NO	YES	YES
0	ENSG00000064601	Galactosialidosis, 256540	YES	NO	NO	YES	NO
0	ENSG00000065328	-	-	-	YES	YES	YES
0	ENSG00000065357	-	-	-	YES	YES	NO
0	ENSG00000065361	-	-	-	YES	NO	YES
0	ENSG00000065559	-	-	-	YES	YES	YES
0	ENSG00000065911	-	-	-	YES	NO	YES
0	ENSG00000065978	-	-	-	NO	YES	YES
0	ENSG00000066044	-	-	-	NO	YES	YES
0	ENSG00000066056	-	-	-	YES	YES	YES
0	ENSG00000066468	-	-	-	YES	YES	YES
0	ENSG00000066827	-	-	-	NO	YES	YES
0	ENSG00000067560	-	-	-	YES	YES	NO
0	ENSG00000067596	-	-	-	YES	YES	NO
0	ENSG00000067715	-	-	-	YES	NO	YES
0	ENSG00000067955	-	-	-	NO	YES	YES
0	ENSG00000068024	-	-	-	YES	NO	YES
0	ENSG00000068078	-	-	-	YES	YES	YES
0	ENSG00000068383	-	-	-	YES	NO	YES
0	ENSG00000068796	-	-	-	YES	NO	YES
0	ENSG00000069702	-	-	-	YES	NO	YES
0	ENSG00000069956	-	-	-	YES	NO	YES
0	ENSG00000070159	-	-	-	YES	YES	NO
0	ENSG00000070193	-	-	-	YES	YES	YES
0	ENSG00000070444	-	-	-	NO	YES	YES
0	ENSG00000070495	-	-	-	NO	YES	YES
0	ENSG00000070501	-	-	-	YES	NO	YES
0	ENSG00000070748	-	-	-	NO	YES	YES
0	ENSG00000070831	-	-	-	NO	YES	YES
0	ENSG00000070950	-	-	-	YES	YES	NO
0	ENSG00000070961	-	-	-	NO	YES	YES
0	ENSG00000071054	-	-	-	YES	NO	YES
0	ENSG00000071189	-	-	-	NO	YES	YES
0	ENSG00000071539	-	-	-	YES	NO	YES
0	ENSG00000072062	-	-	-	YES	NO	YES
0	ENSG00000072274	-	-	-	YES	YES	YES
0	ENSG00000072415	-	-	-	YES	YES	NO
0	ENSG00000072518	-	-	-	YES	NO	YES
0	ENSG00000073282	-	-	-	NO	YES	YES
0	ENSG00000073536	-	-	-	YES	YES	YES
0	ENSG00000073614	-	-	-	YES	NO	YES
0	ENSG00000073712	-	-	-	YES	NO	YES
0	ENSG00000073756	-	-	-	NO	YES	YES
0	ENSG00000074047	-	-	-	YES	YES	YES

0	ENSG00000074201	-	-	-	YES	NO	YES
0	ENSG00000074266	-	-	-	YES	YES	NO
0	ENSG00000074319	-	-	-	YES	NO	YES
0	ENSG00000074590	-	-	-	YES	NO	YES
0	ENSG00000074800	-	-	-	YES	NO	YES
0	ENSG00000074803	-	-	-	NO	YES	YES
0	ENSG00000075213	-	-	-	NO	YES	YES
0	ENSG00000075413	-	-	-	YES	NO	YES
0	ENSG00000075426	-	-	-	YES	NO	YES
0	ENSG00000075618	-	-	-	YES	YES	NO
0	ENSG00000075624	-	-	-	YES	NO	YES
0	ENSG00000075711	-	-	-	YES	YES	YES
0	ENSG00000075891	Optic nerve coloboma with renal disease, 120330	YES	NO	NO	YES	YES
0	ENSG00000076003	-	-	-	YES	NO	YES
0	ENSG00000076201	-	-	-	YES	YES	YES
0	ENSG00000076242	-	-	-	YES	YES	YES
0	ENSG00000076604	-	-	-	YES	NO	YES
0	ENSG00000077044	-	-	-	YES	NO	YES
0	ENSG00000077097	-	-	-	YES	NO	YES
0	ENSG00000077235	-	-	-	YES	NO	YES
0	ENSG00000077279	Lissencephaly, X-linked, 300067; Subcortical laminal heteropia, X-linked, 300067	YES	NO	NO	YES	YES
0	ENSG00000077463	-	-	-	NO	YES	YES
0	ENSG00000077498	-	-	-	NO	YES	YES
0	ENSG00000077782	Pfeiffer syndrome, 101600; Jackson-Weiss sydnrome, 123150	YES	NO	YES	YES	YES
0	ENSG00000078142	-	-	-	YES	NO	YES
0	ENSG00000079102	-	-	-	NO	YES	YES
0	ENSG00000079246	-	-	-	YES	YES	YES
0	ENSG00000079335	-	-	-	YES	YES	NO
0	ENSG00000079616	-	-	-	NO	YES	YES
0	ENSG00000079805	-	-	-	YES	NO	YES
0	ENSG00000079999	-	-	-	NO	YES	YES
0	ENSG00000080493	-	-	-	NO	YES	YES
0	ENSG00000080618	-	-	-	YES	NO	YES
0	ENSG00000080815	-	-	-	NO	YES	YES
0	ENSG00000080824	-	-	-	YES	YES	NO
0	ENSG00000080839	-	-	-	YES	NO	YES
0	ENSG00000080986	-	-	-	YES	YES	NO
0	ENSG00000081237	-	-	-	YES	NO	YES
0	ENSG00000082175	-	-	-	YES	YES	NO
0	ENSG00000082258	-	-	-	YES	NO	YES
0	ENSG00000082293	-	-	-	YES	NO	YES
0	ENSG00000082458	-	-	-	YES	YES	NO
0	ENSG00000082701	-	-	-	YES	NO	YES

0	ENSG00000083720	-	-	-	YES	NO	YES
0	ENSG00000083857	-	-	-	NO	YES	YES
0	ENSG00000084073	-	-	-	NO	YES	YES
0	ENSG00000084754	LCHAD deficiency, 609016; Trifunctional protein deficiency, 609015; HELLP syndrome, maternal, of pregnancy; Fatty liver, acute, of pregnancy	YES	NO	NO	NO	YES
0	ENSG00000085224	-	-	-	YES	NO	YES
0	ENSG00000085563	-	-	-	YES	YES	NO
0	ENSG00000085721	-	-	-	YES	NO	YES
0	ENSG00000085733	-	-	-	YES	NO	YES
0	ENSG00000085741	-	-	-	YES	NO	YES
0	ENSG00000086232	-	-	-	YES	YES	NO
0	ENSG00000087085	-	-	-	NO	YES	YES
0	ENSG00000087087	-	-	-	NO	YES	YES
0	ENSG00000087191	-	-	-	YES	YES	NO
0	ENSG00000087274	-	-	-	NO	YES	YES
0	ENSG00000087460	-	-	-	NO	YES	YES
0	ENSG00000087470	-	-	-	YES	NO	YES
0	ENSG00000087510	-	-	-	YES	NO	YES
0	ENSG00000087586	-	-	-	YES	NO	YES
0	ENSG00000088038	-	-	-	NO	YES	YES
0	ENSG00000088305	Immunodeficiency-centromeric instability-facial anomalies syndrome, 242860	YES	NO	YES	YES	YES
0	ENSG00000088986	-	-	-	YES	YES	NO
0	ENSG00000089225	Holt-Oram syndrome, 142900	YES	NO	YES	NO	YES
0	ENSG00000089280	-	-	-	YES	YES	YES
0	ENSG00000089685	-	-	-	YES	YES	YES
0	ENSG00000090020	-	-	-	NO	YES	YES
0	ENSG00000090273	-	-	-	YES	YES	NO
0	ENSG00000091140	Lipoamide dehydrogenase deficiency, 238331	YES	NO	NO	NO	YES
0	ENSG00000091409	Epidermolysis bullosa, junctional, with pyloric stenosis, 226730	YES	NO	YES	YES	YES
0	ENSG00000091483	Fumarase deficiency, 606812	YES	NO	YES	NO	YES
0	ENSG00000091831	-	-	-	YES	YES	NO
0	ENSG00000092199	-	-	-	YES	NO	YES
0	ENSG00000092295	-	-	-	NO	YES	YES
0	ENSG00000092439	-	-	-	YES	YES	YES
0	ENSG00000092531	-	-	-	YES	NO	YES
0	ENSG00000092621	-	-	-	NO	YES	YES
0	ENSG00000092820	-	-	-	NO	YES	YES
0	ENSG00000092969	-	-	-	YES	YES	YES
0	ENSG00000093009	-	-	-	YES	NO	YES
0	ENSG00000094804	-	-	-	YES	YES	NO
0	ENSG00000094880	-	-	-	YES	YES	NO
0	ENSG00000095015	-	-	-	YES	YES	NO

0	ENSG00000095370	-	-	-	NO	YES	YES
0	ENSG00000096384	-	-	-	YES	NO	YES
0	ENSG00000096696	-	-	-	NO	YES	YES
0	ENSG00000096717	-	-	-	YES	NO	YES
0	ENSG00000096968	-	-	-	YES	YES	YES
0	ENSG00000097007	-	-	-	YES	YES	YES
0	ENSG00000097046	-	-	-	YES	YES	YES
0	ENSG00000099194	-	-	-	YES	YES	YES
0	ENSG00000099804	-	-	-	YES	YES	NO
0	ENSG00000099942	-	-	-	YES	NO	YES
0	ENSG00000099956	-	-	-	NO	YES	YES
0	ENSG00000099991	-	-	-	YES	NO	YES
0	ENSG00000100029	-	-	-	YES	YES	YES
0	ENSG00000100030	-	-	-	YES	YES	YES
0	ENSG00000100033	-	-	-	YES	YES	NO
0	ENSG00000100084	-	-	-	YES	NO	YES
0	ENSG00000100138	-	-	-	YES	YES	NO
0	ENSG00000100146	-	-	-	YES	YES	YES
0	ENSG00000100292	-	-	-	YES	YES	YES
0	ENSG00000100299	Metachromatic leukodystrophy, 250100	YES	NO	NO	YES	NO
0	ENSG00000100324	-	-	-	YES	NO	YES
0	ENSG00000100345	-	-	-	NO	YES	YES
0	ENSG00000100348	-	-	-	YES	NO	YES
0	ENSG00000100393	-	-	-	YES	YES	YES
0	ENSG00000100401	-	-	-	NO	YES	YES
0	ENSG00000100410	-	-	-	YES	YES	YES
0	ENSG00000100412	-	-	-	YES	YES	NO
0	ENSG00000100462	-	-	-	YES	NO	YES
0	ENSG00000100578	-	-	-	YES	YES	YES
0	ENSG00000100596	-	-	-	NO	YES	YES
0	ENSG00000100697	-	-	-	NO	YES	YES
0	ENSG00000100714	-	-	-	YES	NO	YES
0	ENSG00000100764	-	-	-	YES	NO	YES
0	ENSG00000100811	-	-	-	YES	NO	YES
0	ENSG00000100815	-	-	-	NO	YES	YES
0	ENSG00000100823	-	-	-	NO	YES	YES
0	ENSG00000100906	-	-	-	YES	NO	YES
0	ENSG00000101003	-	-	-	YES	NO	YES
0	ENSG00000101115	-	-	-	YES	NO	YES
0	ENSG00000101144	-	-	-	NO	YES	YES
0	ENSG00000101146	-	-	-	YES	YES	YES
0	ENSG00000101190	-	-	-	YES	YES	NO
0	ENSG00000101200	-	-	-	NO	YES	YES
0	ENSG00000101246	-	-	-	NO	YES	YES
0	ENSG00000101266	-	-	-	YES	NO	YES
0	ENSG00000101290	-	-	-	YES	NO	YES

0	ENSG00000101384	-	-	-	YES	YES	YES
0	ENSG00000101489	-	-	-	NO	YES	YES
0	ENSG00000101665	-	-	-	YES	NO	YES
0	ENSG00000101981	Hemophilia B, 306900	YES	NO	NO	NO	YES
0	ENSG00000102145	-	-	-	NO	YES	YES
0	ENSG00000102710	-	-	-	NO	YES	YES
0	ENSG00000102755	-	-	-	YES	NO	YES
0	ENSG00000102786	-	-	-	YES	NO	YES
0	ENSG00000102882	-	-	-	YES	YES	NO
0	ENSG00000102898	-	-	-	YES	YES	NO
0	ENSG00000103035	-	-	-	YES	NO	YES
0	ENSG00000103043	-	-	-	YES	NO	YES
0	ENSG00000103126	-	-	-	NO	YES	YES
0	ENSG00000103197	-	-	-	YES	YES	YES
0	ENSG00000103245	-	-	-	YES	NO	YES
0	ENSG00000103275	-	-	-	NO	YES	YES
0	ENSG00000103423	-	-	-	YES	YES	YES
0	ENSG00000103449	Townes-Brocks syndrome, 107480	YES	NO	NO	NO	YES
0	ENSG00000103479	-	-	-	YES	NO	YES
0	ENSG00000103494	-	-	-	NO	YES	YES
0	ENSG00000103507	-	-	-	YES	NO	YES
0	ENSG00000103510	-	-	-	NO	YES	YES
0	ENSG00000103653	-	-	-	YES	YES	YES
0	ENSG00000103876	Tyrosinemia type I, 276700	YES	NO	NO	YES	YES
0	ENSG00000104064	-	-	-	YES	NO	YES
0	ENSG00000104129	-	-	-	YES	NO	YES
0	ENSG00000104154	-	-	-	NO	YES	YES
0	ENSG00000104313	-	-	-	NO	YES	YES
0	ENSG00000104365	-	-	-	YES	YES	YES
0	ENSG00000104517	-	-	-	YES	NO	YES
0	ENSG00000104763	-	-	-	NO	YES	YES
0	ENSG00000104812	-	-	-	NO	YES	YES
0	ENSG00000104852	-	-	-	YES	YES	NO
0	ENSG00000104856	-	-	-	YES	YES	YES
0	ENSG00000104884	-	-	-	NO	YES	YES
0	ENSG00000105173	-	-	-	YES	YES	NO
0	ENSG00000105202	-	-	-	YES	YES	YES
0	ENSG00000105220	-	-	-	YES	NO	YES
0	ENSG00000105258	-	-	-	YES	YES	NO
0	ENSG00000105325	-	-	-	YES	NO	YES
0	ENSG00000105329	-	-	-	NO	YES	YES
0	ENSG00000105372	Anemia, Diamond-Blackfan, 105650	YES	NO	NO	NO	YES
0	ENSG00000105397	-	-	-	YES	YES	NO
0	ENSG00000105402	-	-	-	NO	YES	YES
0	ENSG00000105426	-	-	-	YES	NO	YES
0	ENSG00000105486	-	-	-	NO	YES	YES

0	ENSG00000105612	-	-	-	NO	YES	YES
0	ENSG00000105698	-	-	-	YES	NO	YES
0	ENSG00000105699	-	-	-	NO	YES	YES
0	ENSG00000105819	-	-	-	YES	NO	YES
0	ENSG00000105976	-	-	-	YES	NO	YES
0	ENSG00000105989	-	-	-	YES	NO	YES
0	ENSG00000105997	-	-	-	NO	YES	YES
0	ENSG00000106004	-	-	-	YES	NO	YES
0	ENSG00000106038	-	-	-	YES	NO	YES
0	ENSG00000106070	-	-	-	YES	YES	YES
0	ENSG00000106459	-	-	-	NO	YES	YES
0	ENSG00000106462	-	-	-	NO	YES	YES
0	ENSG00000106546	-	-	-	YES	YES	YES
0	ENSG00000106571	-	-	-	YES	YES	YES
0	ENSG00000106692	Walker-Warburg syndrome, 236670	YES	NO	NO	NO	YES
0	ENSG00000106799	-	-	-	YES	NO	YES
0	ENSG00000106976	-	-	-	YES	NO	YES
0	ENSG00000107187	-	-	-	NO	YES	YES
0	ENSG00000107263	-	-	-	NO	YES	YES
0	ENSG00000107485	Hypoparathyroidism, sensorineural deafness, and renal dysplasia, 146255	YES	NO	NO	NO	YES
0	ENSG00000107611	-	-	-	YES	NO	YES
0	ENSG00000107643	-	-	-	YES	YES	NO
0	ENSG00000107669	-	-	-	NO	YES	YES
0	ENSG00000107779	-	-	-	YES	YES	YES
0	ENSG00000107831	-	-	-	NO	YES	YES
0	ENSG00000107968	-	-	-	YES	NO	YES
0	ENSG00000107984	-	-	-	NO	YES	YES
0	ENSG00000108010	-	-	-	NO	YES	YES
0	ENSG00000108175	-	-	-	YES	NO	YES
0	ENSG00000108264	-	-	-	YES	YES	NO
0	ENSG00000108306	-	-	-	NO	YES	YES
0	ENSG00000108379	-	-	-	NO	YES	YES
0	ENSG00000108381	Canavan disease, 271900	YES	NO	NO	YES	YES
0	ENSG00000108384	-	-	-	YES	NO	YES
0	ENSG00000108424	-	-	-	YES	NO	YES
0	ENSG00000108576	-	-	-	NO	YES	YES
0	ENSG00000108578	-	-	-	NO	YES	YES
0	ENSG00000108753	-	-	-	NO	YES	YES
0	ENSG00000108821	-	-	-	YES	YES	YES
0	ENSG00000108840	-	-	-	YES	YES	NO
0	ENSG00000108883	-	-	-	YES	YES	NO
0	ENSG00000108946	-	-	-	NO	YES	YES
0	ENSG00000108953	-	-	-	YES	NO	YES
0	ENSG00000108963	-	-	-	YES	NO	YES
0	ENSG00000109320	-	-	-	NO	YES	YES

0	ENSG00000109339	-	-	-	YES	YES	NO
0	ENSG00000109458	-	-	-	YES	YES	YES
0	ENSG00000109501	-	-	-	NO	YES	YES
0	ENSG00000109606	-	-	-	YES	YES	NO
0	ENSG00000109670	-	-	-	NO	YES	YES
0	ENSG00000109705	-	-	-	NO	YES	YES
0	ENSG00000109917	-	-	-	YES	NO	YES
0	ENSG00000110090	CPT deficiency, hepatic, type I, 255120	YES	NO	NO	NO	YES
0	ENSG00000110092	-	-	-	YES	YES	YES
0	ENSG00000110107	-	-	-	YES	YES	YES
0	ENSG00000110172	-	-	-	YES	NO	YES
0	ENSG00000110321	-	-	-	YES	NO	YES
0	ENSG00000110436	-	-	-	NO	YES	YES
0	ENSG00000110536	-	-	-	YES	NO	YES
0	ENSG00000110693	-	-	-	NO	YES	YES
0	ENSG00000110711	-	-	-	NO	YES	YES
0	ENSG00000110713	-	-	-	NO	YES	YES
0	ENSG00000110721	-	-	-	YES	NO	YES
0	ENSG00000111142	-	-	-	NO	YES	YES
0	ENSG00000111145	-	-	-	YES	NO	YES
0	ENSG00000111206	-	-	-	YES	YES	YES
0	ENSG00000111229	-	-	-	YES	NO	YES
0	ENSG00000111276	-	-	-	YES	YES	NO
0	ENSG00000111328	-	-	-	YES	NO	YES
0	ENSG00000111361	-	-	-	YES	YES	NO
0	ENSG00000111424	-	-	-	YES	YES	NO
0	ENSG00000111640	-	-	-	YES	NO	YES
0	ENSG00000111667	-	-	-	YES	NO	YES
0	ENSG00000111799	-	-	-	YES	NO	YES
0	ENSG00000111845	-	-	-	YES	YES	NO
0	ENSG00000111880	-	-	-	YES	YES	NO
0	ENSG00000112033	-	-	-	NO	YES	YES
0	ENSG00000112081	-	-	-	YES	NO	YES
0	ENSG00000112096	-	-	-	YES	YES	YES
0	ENSG00000112118	-	-	-	YES	NO	YES
0	ENSG00000112242	-	-	-	YES	NO	YES
0	ENSG00000112294	-	-	-	NO	YES	YES
0	ENSG00000112312	-	-	-	YES	YES	YES
0	ENSG00000112333	-	-	-	YES	YES	NO
0	ENSG00000112357	Rhizomelic chondrodysplasia punctata, type 1, 215100	YES	NO	NO	YES	YES
0	ENSG00000112531	-	-	-	NO	YES	YES
0	ENSG00000112559	-	-	-	NO	YES	YES
0	ENSG00000112578	-	-	-	YES	NO	YES
0	ENSG00000112592	-	-	-	NO	YES	YES
0	ENSG00000112640	-	-	-	YES	NO	YES

0	ENSG00000112658	-	-	-	YES	NO	YES
0	ENSG00000112715	-	-	-	YES	YES	YES
0	ENSG00000113070	-	-	-	NO	YES	YES
0	ENSG00000113083	-	-	-	NO	YES	YES
0	ENSG00000113161	-	-	-	YES	NO	YES
0	ENSG00000113448	-	-	-	YES	NO	YES
0	ENSG00000113494	-	-	-	YES	YES	NO
0	ENSG00000113522	-	-	-	YES	YES	YES
0	ENSG00000113558	-	-	-	YES	YES	NO
0	ENSG00000113575	-	-	-	YES	NO	YES
0	ENSG00000113578	-	-	-	YES	YES	NO
0	ENSG00000113580	Cortisol resistance, 138040	NO	YES	YES	YES	YES
0	ENSG00000113643	-	-	-	YES	YES	NO
0	ENSG00000113658	-	-	-	YES	YES	YES
0	ENSG00000113721	-	-	-	YES	YES	YES
0	ENSG00000113810	-	-	-	YES	YES	NO
0	ENSG00000113916	-	-	-	NO	YES	YES
0	ENSG00000114270	-	-	-	NO	YES	YES
0	ENSG00000114346	-	-	-	YES	NO	YES
0	ENSG00000114739	-	-	-	YES	NO	YES
0	ENSG00000115020	-	-	-	YES	NO	YES
0	ENSG00000115138	-	-	-	NO	YES	YES
0	ENSG00000115170	-	-	-	YES	NO	YES
0	ENSG00000115241	-	-	-	YES	NO	YES
0	ENSG00000115306	-	-	-	NO	YES	YES
0	ENSG00000115414	-	-	-	YES	YES	YES
0	ENSG00000115415	-	-	-	YES	YES	YES
0	ENSG00000115486	Vitamin K-dependent coagulation defect, 277450	YES	NO	NO	NO	YES
0	ENSG00000115524	-	-	-	YES	NO	YES
0	ENSG00000115657	-	-	-	YES	YES	NO
0	ENSG00000115718	Thrombophilia due to protein C deficiency, 176860; neonatal purpura fulminans	YES	NO	NO	YES	YES
0	ENSG00000115738	-	-	-	YES	YES	YES
0	ENSG00000115758	-	-	-	NO	YES	YES
0	ENSG00000115760	-	-	-	YES	NO	YES
0	ENSG00000115947	-	-	-	YES	YES	NO
0	ENSG00000115966	-	-	-	YES	NO	YES
0	ENSG00000116014	Hypogonadotropic hypogonadism, 146110	NO	YES	NO	NO	YES
0	ENSG00000116017	-	-	-	YES	NO	YES
0	ENSG00000116030	-	-	-	YES	YES	YES
0	ENSG00000116096	-	-	-	NO	YES	YES
0	ENSG00000116132	-	-	-	NO	YES	YES
0	ENSG00000116133	-	-	-	NO	YES	YES
0	ENSG00000116266	-	-	-	YES	NO	YES
0	ENSG00000116285	-	-	-	NO	YES	YES

0	ENSG00000116478	-	-	-	YES	YES	YES
0	ENSG00000116679	-	-	-	YES	NO	YES
0	ENSG00000116704	-	-	-	YES	YES	YES
0	ENSG00000116809	-	-	-	YES	NO	YES
0	ENSG00000116833	-	-	-	YES	YES	YES
0	ENSG00000116984	-	-	-	NO	YES	YES
0	ENSG00000117054	Acyl-CoA dehydrogenase, median-chain, deficiency of, 201450	YES	NO	NO	YES	YES
0	ENSG00000117298	Hirschsprung disease, 142623	YES	NO	NO	YES	YES
0	ENSG00000117305	-	-	-	NO	YES	YES
0	ENSG00000117394	-	-	-	NO	YES	YES
0	ENSG00000117399	-	-	-	YES	NO	YES
0	ENSG00000117505	-	-	-	YES	YES	NO
0	ENSG00000117595	-	-	-	NO	YES	YES
0	ENSG00000117601	-	-	-	NO	YES	YES
0	ENSG00000117682	-	-	-	YES	YES	NO
0	ENSG00000117751	-	-	-	YES	NO	YES
0	ENSG00000117984	Congenital neuronal ceroid lipofuscinosis 10, 610127	YES	NO	NO	YES	NO
0	ENSG00000118046	Peutz-Jeghers syndrome, 175200	YES	NO	NO	YES	YES
0	ENSG00000118260	-	-	-	NO	YES	YES
0	ENSG00000118402	-	-	-	NO	YES	YES
0	ENSG00000118513	-	-	-	YES	YES	YES
0	ENSG00000118523	-	-	-	YES	NO	YES
0	ENSG00000118526	-	-	-	YES	NO	YES
0	ENSG00000118640	-	-	-	NO	YES	YES
0	ENSG00000118655	-	-	-	YES	NO	YES
0	ENSG00000118762	-	-	-	NO	YES	YES
0	ENSG00000119121	-	-	-	YES	NO	YES
0	ENSG00000119318	-	-	-	YES	NO	YES
0	ENSG00000119383	-	-	-	YES	YES	NO
0	ENSG00000119508	-	-	-	YES	NO	YES
0	ENSG00000119523	-	-	-	YES	YES	NO
0	ENSG00000119681	-	-	-	YES	NO	YES
0	ENSG00000119699	-	-	-	YES	NO	YES
0	ENSG00000119772	-	-	-	NO	YES	YES
0	ENSG00000119899	-	-	-	NO	YES	YES
0	ENSG00000120156	-	-	-	YES	NO	YES
0	ENSG00000120251	-	-	-	NO	YES	YES
0	ENSG00000120314	-	-	-	YES	NO	YES
0	ENSG00000120708	-	-	-	YES	YES	NO
0	ENSG00000120756	-	-	-	YES	YES	NO
0	ENSG00000120868	-	-	-	YES	YES	YES
0	ENSG00000120885	-	-	-	YES	YES	NO
0	ENSG00000121022	-	-	-	NO	YES	YES
0	ENSG00000121691	-	-	-	YES	YES	NO

0	ENSG00000121774	-	-	-	YES	NO	YES
0	ENSG00000121858	-	-	-	YES	YES	NO
0	ENSG00000121879	-	-	-	YES	YES	YES
0	ENSG00000121966	-	-	-	NO	YES	YES
0	ENSG00000122025	-	-	-	YES	YES	NO
0	ENSG00000122257	-	-	-	YES	NO	YES
0	ENSG00000122367	-	-	-	NO	YES	YES
0	ENSG00000122691	-	-	-	NO	YES	YES
0	ENSG00000122756	-	-	-	NO	YES	YES
0	ENSG00000122877	-	-	-	YES	NO	YES
0	ENSG00000122966	-	-	-	YES	NO	YES
0	ENSG00000123154	-	-	-	YES	YES	YES
0	ENSG00000123307	-	-	-	YES	NO	YES
0	ENSG00000123374	-	-	-	YES	YES	YES
0	ENSG00000123405	-	-	-	NO	YES	YES
0	ENSG00000123454	-	-	-	NO	YES	YES
0	ENSG00000123505	-	-	-	YES	YES	YES
0	ENSG00000123560	-	-	-	NO	YES	YES
0	ENSG00000123737	-	-	-	YES	YES	NO
0	ENSG00000123908	-	-	-	YES	NO	YES
0	ENSG00000124151	-	-	-	YES	NO	YES
0	ENSG00000124205	-	-	-	NO	YES	YES
0	ENSG00000124253	-	-	-	YES	NO	YES
0	ENSG00000124479	-	-	-	YES	YES	NO
0	ENSG00000124535	-	-	-	YES	YES	NO
0	ENSG00000124615	Molybdenum cofactor deficiency, type A, 252150	YES	NO	NO	NO	YES
0	ENSG00000124766	-	-	-	YES	NO	YES
0	ENSG00000125084	-	-	-	YES	NO	YES
0	ENSG00000125347	-	-	-	YES	YES	NO
0	ENSG00000125398	Campomelic dysplasia with autosomal sex reversal, 114290	YES	NO	NO	YES	YES
0	ENSG00000125454	Amish lethal microcephaly, 607196	YES	NO	NO	YES	YES
0	ENSG00000125630	-	-	-	YES	NO	YES
0	ENSG00000125798	-	-	-	NO	YES	YES
0	ENSG00000125845	-	-	-	NO	YES	YES
0	ENSG00000125863	Bardet-Biedl syndrome, 209900; McKusick-Kaufman syndrome, 236700	YES	NO	NO	YES	YES
0	ENSG00000125877	-	-	-	YES	NO	YES
0	ENSG00000125885	-	-	-	YES	YES	NO
0	ENSG00000125952	-	-	-	YES	YES	YES
0	ENSG00000125977	-	-	-	YES	NO	YES
0	ENSG00000126215	-	-	-	YES	YES	NO
0	ENSG00000126216	-	-	-	YES	YES	NO
0	ENSG00000126457	-	-	-	YES	NO	YES
0	ENSG00000126581	-	-	-	NO	YES	YES

0	ENSG00000126653	-	-	-	YES	NO	YES
0	ENSG00000127528	-	-	-	NO	YES	YES
0	ENSG00000127603	-	-	-	NO	YES	YES
0	ENSG00000127616	-	-	-	YES	YES	YES
0	ENSG00000127947	-	-	-	YES	NO	YES
0	ENSG00000127948	POR deficiency, 201750; Antley-Bixler syndrome, 207410	YES	YES	NO	YES	YES
0	ENSG00000128052	-	-	-	YES	NO	YES
0	ENSG00000128191	-	-	-	YES	NO	YES
0	ENSG00000128683	-	-	-	NO	YES	YES
0	ENSG00000128791	-	-	-	YES	NO	YES
0	ENSG00000128829	-	-	-	YES	YES	YES
0	ENSG00000128917	-	-	-	NO	YES	YES
0	ENSG00000128918	-	-	-	NO	YES	YES
0	ENSG00000129116	-	-	-	NO	YES	YES
0	ENSG00000129351	-	-	-	NO	YES	YES
0	ENSG00000129562	-	-	-	NO	YES	YES
0	ENSG00000129691	-	-	-	YES	NO	YES
0	ENSG00000129757	-	-	-	YES	NO	YES
0	ENSG00000130385	Ovarian dysgenesis 2, 300510	NO	YES	YES	NO	NO
0	ENSG00000130489	-	-	-	YES	YES	YES
0	ENSG00000130635	-	-	-	YES	NO	YES
0	ENSG00000130669	-	-	-	NO	YES	YES
0	ENSG00000130675	Currarino syndrome, 176450	YES	NO	NO	NO	YES
0	ENSG00000130707	Citrullinemia, 215700	YES	NO	NO	NO	YES
0	ENSG00000130714	Walker-Warburg syndrome, 236670	YES	NO	NO	YES	YES
0	ENSG00000130726	-	-	-	YES	NO	YES
0	ENSG00000130816	-	-	-	YES	YES	YES
0	ENSG00000130826	-	-	-	YES	YES	NO
0	ENSG00000131051	-	-	-	YES	NO	YES
0	ENSG00000131269	-	-	-	YES	NO	YES
0	ENSG00000131437	-	-	-	NO	YES	YES
0	ENSG00000131446	-	-	-	NO	YES	YES
0	ENSG00000131482	-	-	-	YES	YES	NO
0	ENSG00000131558	-	-	-	NO	YES	YES
0	ENSG00000131620	-	-	-	YES	NO	YES
0	ENSG00000131759	-	-	-	YES	NO	YES
0	ENSG00000131828	Pyruvate dehydrogenase deficiency, 312170	YES	NO	NO	NO	YES
0	ENSG00000131899	-	-	-	YES	YES	YES
0	ENSG00000132002	-	-	-	YES	YES	NO
0	ENSG00000132130	-	-	-	NO	YES	YES
0	ENSG00000132142	-	-	-	YES	YES	YES
0	ENSG00000132155	-	-	-	YES	NO	YES
0	ENSG00000132170	-	-	-	YES	YES	YES
0	ENSG00000132341	-	-	-	YES	YES	NO
0	ENSG00000132383	-	-	-	NO	YES	YES

0	ENSG00000132437	-	-	-	NO	YES	YES
0	ENSG00000132470	Epidermolysis bullosa, junctional, with pyloric atresia, 226730; Epidermolysis bullosa, generalized atrophic benign, 226650	YES	NO	NO	YES	YES
0	ENSG00000132639	-	-	-	NO	YES	YES
0	ENSG00000132646	-	-	-	YES	YES	YES
0	ENSG00000132688	-	-	-	NO	YES	YES
0	ENSG00000132780	-	-	-	YES	NO	YES
0	ENSG00000132792	-	-	-	YES	YES	NO
0	ENSG00000132842	-	-	-	YES	NO	YES
0	ENSG00000132964	-	-	-	YES	NO	YES
0	ENSG00000133110	-	-	-	NO	YES	YES
0	ENSG00000133112	-	-	-	YES	NO	YES
0	ENSG00000133392	-	-	-	NO	YES	YES
0	ENSG00000133703	-	-	-	YES	NO	YES
0	ENSG00000133835	D-bifunctional protein deficiency, 261515	YES	NO	NO	NO	YES
0	ENSG00000133895	-	-	-	YES	YES	YES
0	ENSG00000133961	-	-	-	YES	NO	YES
0	ENSG00000134001	-	-	-	YES	YES	YES
0	ENSG00000134057	-	-	-	YES	YES	YES
0	ENSG00000134250	-	-	-	NO	YES	YES
0	ENSG00000134259	-	-	-	NO	YES	YES
0	ENSG00000134308	-	-	-	YES	NO	YES
0	ENSG00000134318	-	-	-	YES	NO	YES
0	ENSG00000134323	-	-	-	NO	YES	YES
0	ENSG00000134363	-	-	-	NO	YES	YES
0	ENSG00000134532	-	-	-	YES	NO	YES
0	ENSG00000134575	Acid phosphatase deficiency, 200950	YES	NO	YES	NO	NO
0	ENSG00000134595	-	-	-	YES	NO	YES
0	ENSG00000134853	-	-	-	YES	YES	YES
0	ENSG00000134899	Xeroderma pigmentosum, group G, 278780	YES	NO	YES	NO	YES
0	ENSG00000134910	-	-	-	YES	YES	NO
0	ENSG00000134954	-	-	-	YES	NO	YES
0	ENSG00000134982	Gardner syndrome; Adenomatous polyposis coli; Colorectal cancer, 114500; Desmoid disease, hereditary, 135290; Medulloblastoma, 155255; Turcot syndrome, 276300	YES	NO	YES	YES	YES
0	ENSG00000135002	-	-	-	YES	NO	YES
0	ENSG00000135047	-	-	-	YES	NO	YES
0	ENSG00000135334	-	-	-	YES	NO	YES
0	ENSG00000135341	-	-	-	YES	NO	YES
0	ENSG00000135423	-	-	-	YES	YES	NO
0	ENSG00000135476	-	-	-	YES	YES	YES
0	ENSG00000135547	-	-	-	NO	YES	YES
0	ENSG00000135679	-	-	-	YES	YES	YES
0	ENSG00000135766	-	-	-	NO	YES	YES

0	ENSG00000135821	-	-	-	YES	YES	YES
0	ENSG00000135829	-	-	-	YES	NO	YES
0	ENSG00000135903	-	-	-	NO	YES	YES
0	ENSG00000135945	-	-	-	YES	YES	YES
0	ENSG00000135960	-	-	-	NO	YES	YES
0	ENSG00000136068	-	-	-	NO	YES	YES
0	ENSG00000136160	Hirschsprung disease-2, 600155	YES	NO	NO	YES	YES
0	ENSG00000136238	-	-	-	YES	NO	YES
0	ENSG00000136352	-	-	-	NO	YES	YES
0	ENSG00000136448	-	-	-	NO	YES	YES
0	ENSG00000136450	-	-	-	YES	NO	YES
0	ENSG00000136527	-	-	-	YES	NO	YES
0	ENSG00000136574	-	-	-	NO	YES	YES
0	ENSG00000136630	-	-	-	NO	YES	YES
0	ENSG00000136717	-	-	-	YES	YES	YES
0	ENSG00000136807	-	-	-	YES	YES	NO
0	ENSG00000136811	-	-	-	YES	NO	YES
0	ENSG00000136826	-	-	-	YES	NO	YES
0	ENSG00000136868	-	-	-	NO	YES	YES
0	ENSG00000136931	Sex reversal, XY, with adrenal failure, 184757	NO	YES	YES	NO	YES
0	ENSG00000136936	-	-	-	YES	YES	YES
0	ENSG00000136960	-	-	-	NO	YES	YES
0	ENSG00000136997	-	-	-	YES	YES	YES
0	ENSG00000137076	-	-	-	YES	NO	YES
0	ENSG00000137193	-	-	-	YES	YES	NO
0	ENSG00000137203	-	-	-	YES	NO	YES
0	ENSG00000137273	-	-	-	YES	NO	YES
0	ENSG00000137601	-	-	-	YES	NO	YES
0	ENSG00000137710	-	-	-	YES	YES	NO
0	ENSG00000137801	-	-	-	YES	NO	YES
0	ENSG00000137807	-	-	-	YES	YES	NO
0	ENSG00000137845	-	-	-	NO	YES	YES
0	ENSG00000137869	-	-	-	YES	YES	NO
0	ENSG00000138031	-	-	-	NO	YES	YES
0	ENSG00000138039	Pseudohermaphroditism, male, with Leydig cell hypoplasia; Hypogonadotropic hypogonadism; Micropenis; Leydig cell adenoma, with precocious puberty; Precocious puberty, male, 176410	NO	YES	NO	YES	NO
0	ENSG00000138061	-	-	-	YES	YES	NO
0	ENSG00000138083	Holoprosencephaly-2, 157170	YES	NO	NO	NO	YES
0	ENSG00000138160	-	-	-	YES	NO	YES
0	ENSG00000138175	-	-	-	YES	NO	YES
0	ENSG00000138376	-	-	-	YES	NO	YES
0	ENSG00000138385	-	-	-	NO	YES	YES
0	ENSG00000138435	-	-	-	NO	YES	YES

0	ENSG00000138448	-	-	-	YES	NO	YES
0	ENSG00000138449	-	-	-	NO	YES	YES
0	ENSG00000138604	-	-	-	NO	YES	YES
0	ENSG00000138685	-	-	-	YES	YES	NO
0	ENSG00000138696	Chondrodysplasia, acromesomelic, with genital anomalies, 609441	NO	YES	YES	NO	NO
0	ENSG00000138778	-	-	-	YES	NO	YES
0	ENSG00000138798	-	-	-	YES	YES	NO
0	ENSG00000138802	-	-	-	NO	YES	YES
0	ENSG00000138823	-	-	-	NO	YES	YES
0	ENSG00000139083	-	-	-	NO	YES	YES
0	ENSG00000139174	-	-	-	YES	NO	YES
0	ENSG00000139219	-	-	-	NO	YES	YES
0	ENSG00000139318	-	-	-	YES	NO	YES
0	ENSG00000139352	-	-	-	NO	YES	YES
0	ENSG00000139514	-	-	-	YES	NO	YES
0	ENSG00000139549	Partial gonadal dysgenesis with minifascicular neuropathy, 607080	NO	YES	YES	NO	NO
0	ENSG00000139567	-	-	-	YES	YES	YES
0	ENSG00000139618	-	-	-	YES	YES	YES
0	ENSG00000139625	-	-	-	YES	NO	YES
0	ENSG00000139631	-	-	-	YES	YES	NO
0	ENSG00000139687	-	-	-	YES	YES	YES
0	ENSG00000139842	-	-	-	NO	YES	YES
0	ENSG00000140105	-	-	-	YES	YES	NO
0	ENSG00000140326	-	-	-	YES	YES	YES
0	ENSG00000140416	-	-	-	YES	YES	YES
0	ENSG00000140443	-	-	-	YES	NO	YES
0	ENSG00000140521	Alpers syndrome, 203700	YES	NO	NO	YES	YES
0	ENSG00000140538	-	-	-	YES	NO	YES
0	ENSG00000140564	-	-	-	YES	YES	YES
0	ENSG00000140992	-	-	-	YES	NO	YES
0	ENSG00000141027	-	-	-	NO	YES	YES
0	ENSG00000141030	-	-	-	YES	NO	YES
0	ENSG00000141378	-	-	-	YES	NO	YES
0	ENSG00000141448	-	-	-	NO	YES	YES
0	ENSG00000141510	Colorectal cancer, 114500; Esophageal cancer, 133239; Lung cancer, 211980; Horoid plexus papilloma, 260500; Li-Fraumeni syndrome, 151623	YES	NO	YES	YES	YES
0	ENSG00000141551	-	-	-	YES	NO	YES
0	ENSG00000141564	-	-	-	NO	YES	YES
0	ENSG00000141646	-	-	-	YES	YES	YES
0	ENSG00000141720	-	-	-	YES	NO	YES
0	ENSG00000141736	-	-	-	YES	YES	YES
0	ENSG00000141837	-	-	-	NO	YES	YES

0	ENSG00000141867	-	-	-	YES	YES	YES
0	ENSG00000142168	-	-	-	YES	YES	NO
0	ENSG00000142192	-	-	-	YES	YES	NO
0	ENSG00000142208	-	-	-	YES	YES	YES
0	ENSG00000142731	-	-	-	YES	NO	YES
0	ENSG00000142798	Silverman-Handmaker type of dyssegmental dysplasia, 224410	YES	NO	NO	YES	YES
0	ENSG00000142867	-	-	-	YES	NO	YES
0	ENSG00000142871	-	-	-	YES	NO	YES
0	ENSG00000142949	-	-	-	YES	YES	NO
0	ENSG00000143140	-	-	-	YES	NO	YES
0	ENSG00000143256	-	-	-	YES	NO	YES
0	ENSG00000143337	-	-	-	YES	NO	YES
0	ENSG00000143379	-	-	-	YES	YES	YES
0	ENSG00000143384	-	-	-	YES	YES	NO
0	ENSG00000143437	-	-	-	NO	YES	YES
0	ENSG00000143476	-	-	-	NO	YES	YES
0	ENSG00000143799	-	-	-	YES	YES	YES
0	ENSG00000143801	-	-	-	YES	YES	NO
0	ENSG00000143815	-	-	-	NO	YES	YES
0	ENSG00000143816	-	-	-	YES	NO	YES
0	ENSG00000143839	-	-	-	NO	YES	YES
0	ENSG00000143858	-	-	-	NO	YES	YES
0	ENSG00000143952	-	-	-	YES	NO	YES
0	ENSG00000143995	-	-	-	YES	YES	YES
0	ENSG00000144028	-	-	-	YES	NO	YES
0	ENSG00000144381	-	-	-	YES	YES	YES
0	ENSG00000144452	-	-	-	NO	YES	YES
0	ENSG00000144668	-	-	-	NO	YES	YES
0	ENSG00000144744	-	-	-	YES	NO	YES
0	ENSG00000145012	-	-	-	NO	YES	YES
0	ENSG00000145386	-	-	-	YES	YES	YES
0	ENSG00000145604	-	-	-	YES	YES	NO
0	ENSG00000145632	-	-	-	YES	NO	YES
0	ENSG00000145681	-	-	-	NO	YES	YES
0	ENSG00000145715	-	-	-	YES	NO	YES
0	ENSG00000145730	-	-	-	YES	YES	YES
0	ENSG00000145741	-	-	-	YES	YES	YES
0	ENSG00000145888	-	-	-	YES	NO	YES
0	ENSG00000145907	-	-	-	YES	NO	YES
0	ENSG00000146085	-	-	-	NO	YES	YES
0	ENSG00000146247	-	-	-	YES	NO	YES
0	ENSG00000146648	-	-	-	YES	YES	YES
0	ENSG00000147044	-	-	-	YES	YES	YES
0	ENSG00000147099	-	-	-	YES	NO	YES
0	ENSG00000147133	-	-	-	YES	YES	NO

0	ENSG00000147155	-	-	-	NO	YES	YES
0	ENSG00000147316	-	-	-	YES	YES	YES
0	ENSG00000147465	Lipoid adrenal hyperplasia, 201710	YES	NO	NO	NO	YES
0	ENSG00000147571	Autosomal recessive hypothalamic corticotropin deficiency, 122560	YES	NO	YES	YES	NO
0	ENSG00000147601	-	-	-	YES	NO	YES
0	ENSG00000148053	-	-	-	YES	YES	YES
0	ENSG00000148154	-	-	-	NO	YES	YES
0	ENSG00000148180	-	-	-	YES	NO	YES
0	ENSG00000148200	-	-	-	YES	NO	YES
0	ENSG00000148400	-	-	-	NO	YES	YES
0	ENSG00000148408	-	-	-	NO	YES	YES
0	ENSG00000148584	-	-	-	NO	YES	YES
0	ENSG00000148737	-	-	-	YES	NO	YES
0	ENSG00000148795	-	-	-	YES	NO	YES
0	ENSG00000148926	-	-	-	NO	YES	YES
0	ENSG00000149177	-	-	-	YES	NO	YES
0	ENSG00000149187	-	-	-	NO	YES	YES
0	ENSG00000149257	-	-	-	YES	NO	YES
0	ENSG00000149311	T-cell prolymphocytic leukemia, sporadic; Lymphoma, B-cell non-Hodgkin, somatic; Lymphoma, mantel cell; Ataxia-telangiectasia, 208900	NO	YES	NO	YES	NO
0	ENSG00000149480	-	-	-	NO	YES	YES
0	ENSG00000149554	-	-	-	YES	YES	YES
0	ENSG00000149923	-	-	-	YES	YES	YES
0	ENSG00000149968	-	-	-	YES	YES	NO
0	ENSG00000150093	-	-	-	NO	YES	YES
0	ENSG00000150347	-	-	-	YES	NO	YES
0	ENSG00000150630	-	-	-	YES	NO	YES
0	ENSG00000150787	-	-	-	NO	YES	YES
0	ENSG00000150907	-	-	-	YES	NO	YES
0	ENSG00000151247	-	-	-	YES	YES	NO
0	ENSG00000151414	-	-	-	YES	NO	YES
0	ENSG00000151623	-	-	-	YES	YES	YES
0	ENSG00000151657	-	-	-	YES	YES	NO
0	ENSG00000151694	-	-	-	NO	YES	YES
0	ENSG00000151702	-	-	-	YES	NO	YES
0	ENSG00000151892	-	-	-	NO	YES	YES
0	ENSG00000151914	-	-	-	NO	YES	YES
0	ENSG00000151923	-	-	-	NO	YES	YES
0	ENSG00000152332	-	-	-	YES	YES	NO
0	ENSG00000152661	-	-	-	YES	YES	YES
0	ENSG00000152804	-	-	-	NO	YES	YES
0	ENSG00000152942	-	-	-	YES	NO	YES
0	ENSG00000152944	-	-	-	YES	NO	YES

0	ENSG00000153071	-	-	-	NO	YES	YES
0	ENSG00000153147	-	-	-	YES	NO	YES
0	ENSG00000153162	-	-	-	YES	YES	NO
0	ENSG00000153187	-	-	-	YES	NO	YES
0	ENSG00000153250	-	-	-	YES	NO	YES
0	ENSG00000153879	-	-	-	YES	NO	YES
0	ENSG00000153904	-	-	-	NO	YES	YES
0	ENSG00000153944	-	-	-	YES	NO	YES
0	ENSG00000154188	-	-	-	NO	YES	YES
0	ENSG00000154229	-	-	-	YES	YES	NO
0	ENSG00000154473	-	-	-	YES	YES	YES
0	ENSG00000154727	-	-	-	YES	NO	YES
0	ENSG00000154767	-	-	-	YES	YES	NO
0	ENSG00000155130	-	-	-	YES	YES	YES
0	ENSG00000155657	-	-	-	YES	YES	YES
0	ENSG00000155980	-	-	-	YES	NO	YES
0	ENSG00000156110	-	-	-	YES	YES	YES
0	ENSG00000156427	-	-	-	YES	NO	YES
0	ENSG00000156502	-	-	-	NO	YES	YES
0	ENSG00000156709	-	-	-	NO	YES	YES
0	ENSG00000156925	Heterotaxy, X-linked visceral, 306955	YES	NO	NO	NO	YES
0	ENSG00000156970	-	-	-	YES	NO	YES
0	ENSG00000157005	-	-	-	YES	YES	NO
0	ENSG00000157036	-	-	-	YES	YES	NO
0	ENSG00000157106	-	-	-	YES	YES	NO
0	ENSG00000157168	-	-	-	YES	NO	YES
0	ENSG00000157216	-	-	-	NO	YES	YES
0	ENSG00000157227	-	-	-	YES	NO	YES
0	ENSG00000157404	-	-	-	YES	NO	YES
0	ENSG00000157450	-	-	-	NO	YES	YES
0	ENSG00000157456	-	-	-	YES	NO	YES
0	ENSG00000157540	-	-	-	YES	YES	YES
0	ENSG00000157601	-	-	-	YES	YES	NO
0	ENSG00000157764	-	-	-	YES	NO	YES
0	ENSG00000157766	-	-	-	YES	YES	YES
0	ENSG00000158125	-	-	-	NO	YES	YES
0	ENSG00000158195	-	-	-	YES	YES	YES
0	ENSG00000158290	-	-	-	YES	YES	NO
0	ENSG00000158615	-	-	-	YES	NO	YES
0	ENSG00000158813	-	-	-	NO	YES	YES
0	ENSG00000158828	-	-	-	YES	YES	NO
0	ENSG00000158955	-	-	-	NO	YES	YES
0	ENSG00000159164	-	-	-	NO	YES	YES
0	ENSG00000159216	-	-	-	YES	YES	YES
0	ENSG00000159307	-	-	-	YES	NO	YES
0	ENSG00000159352	-	-	-	YES	NO	YES

0	ENSG00000159459	Johanson-Blizzard syndrome, 243800	YES	NO	NO	YES	NO
0	ENSG00000159640	-	-	-	NO	YES	YES
0	ENSG00000159720	-	-	-	YES	NO	YES
0	ENSG00000159723	-	-	-	NO	YES	YES
0	ENSG00000159921	-	-	-	YES	NO	YES
0	ENSG00000160145	-	-	-	YES	YES	NO
0	ENSG00000160199	-	-	-	NO	YES	YES
0	ENSG00000160200	-	-	-	NO	YES	YES
0	ENSG00000160211	-	-	-	NO	YES	YES
0	ENSG00000160285	-	-	-	YES	YES	NO
0	ENSG00000160294	-	-	-	YES	NO	YES
0	ENSG00000160307	-	-	-	YES	YES	NO
0	ENSG00000160323	Atypical hemolytic uremic syndrome, 235400; Congenital thrombotic thrombocytopenic purpura, 274150	YES	NO	NO	YES	NO
0	ENSG00000160691	-	-	-	YES	YES	YES
0	ENSG00000160710	-	-	-	NO	YES	YES
0	ENSG00000160801	-	-	-	NO	YES	YES
0	ENSG00000160973	-	-	-	YES	NO	YES
0	ENSG00000161021	Leukotriene C4 synthase deficiency, 246530	YES	NO	NO	NO	YES
0	ENSG00000161203	-	-	-	YES	NO	YES
0	ENSG00000162063	-	-	-	YES	NO	YES
0	ENSG00000162298	-	-	-	YES	YES	YES
0	ENSG00000162407	-	-	-	YES	NO	YES
0	ENSG00000162409	-	-	-	YES	YES	NO
0	ENSG00000162434	-	-	-	YES	NO	YES
0	ENSG00000162551	-	-	-	NO	YES	YES
0	ENSG00000162607	-	-	-	YES	NO	YES
0	ENSG00000162736	-	-	-	NO	YES	YES
0	ENSG00000162775	-	-	-	NO	YES	YES
0	ENSG00000162909	-	-	-	NO	YES	YES
0	ENSG00000162992	-	-	-	YES	YES	YES
0	ENSG00000163064	-	-	-	NO	YES	YES
0	ENSG00000163161	-	-	-	YES	YES	YES
0	ENSG00000163217	-	-	-	YES	NO	YES
0	ENSG00000163251	-	-	-	YES	NO	YES
0	ENSG00000163347	-	-	-	YES	NO	YES
0	ENSG00000163348	-	-	-	YES	NO	YES
0	ENSG00000163435	-	-	-	YES	NO	YES
0	ENSG00000163482	-	-	-	NO	YES	YES
0	ENSG00000163485	-	-	-	YES	YES	NO
0	ENSG00000163501	-	-	-	NO	YES	YES
0	ENSG00000163513	-	-	-	YES	YES	YES
0	ENSG00000163558	-	-	-	YES	NO	YES
0	ENSG00000163629	-	-	-	YES	YES	NO
0	ENSG00000163785	-	-	-	YES	NO	YES

0	ENSG00000163931	-	-	-	NO	YES	YES
0	ENSG00000163939	-	-	-	YES	NO	YES
0	ENSG00000164066	-	-	-	NO	YES	YES
0	ENSG00000164107	-	-	-	NO	YES	YES
0	ENSG00000164109	-	-	-	YES	NO	YES
0	ENSG00000164305	-	-	-	YES	YES	YES
0	ENSG00000164327	-	-	-	YES	YES	YES
0	ENSG00000164442	-	-	-	NO	YES	YES
0	ENSG00000164506	-	-	-	NO	YES	YES
0	ENSG00000164690	-	-	-	NO	YES	YES
0	ENSG00000164733	-	-	-	YES	YES	NO
0	ENSG00000164751	-	-	-	NO	YES	YES
0	ENSG00000164754	-	-	-	YES	YES	YES
0	ENSG00000164761	-	-	-	NO	YES	YES
0	ENSG00000164953	-	-	-	NO	YES	YES
0	ENSG00000164985	-	-	-	YES	NO	YES
0	ENSG00000165025	-	-	-	YES	NO	YES
0	ENSG00000165060	-	-	-	YES	YES	YES
0	ENSG00000165195	-	-	-	NO	YES	YES
0	ENSG00000165240	-	-	-	NO	YES	YES
0	ENSG00000165271	-	-	-	YES	YES	NO
0	ENSG00000165280	-	-	-	YES	NO	YES
0	ENSG00000165392	-	-	-	NO	YES	YES
0	ENSG00000165458	-	-	-	YES	YES	YES
0	ENSG00000165462	-	-	-	NO	YES	YES
0	ENSG00000165474	-	-	-	YES	YES	YES
0	ENSG00000165494	-	-	-	YES	YES	NO
0	ENSG00000165556	-	-	-	NO	YES	YES
0	ENSG00000165588	-	-	-	NO	YES	YES
0	ENSG00000165699	-	-	-	NO	YES	YES
0	ENSG00000165702	-	-	-	YES	YES	YES
0	ENSG00000165704	-	-	-	YES	YES	NO
0	ENSG00000165731	Multiple endocrine neoplasia IIA, 171400; Medullary thyroid carcinoma, 155240; Multiple endocrine neoplasia IIB, 162300; Hirschsprung disease, 142623	YES	NO	NO	NO	YES
0	ENSG00000165916	-	-	-	YES	NO	YES
0	ENSG00000165917	-	-	-	NO	YES	YES
0	ENSG00000165970	-	-	-	NO	YES	YES
0	ENSG00000165973	-	-	-	NO	YES	YES
0	ENSG00000166147	-	-	-	NO	YES	YES
0	ENSG00000166169	-	-	-	YES	YES	YES
0	ENSG00000166200	-	-	-	NO	YES	YES
0	ENSG00000166311	Niemann-Pick disease, type A, 257200; Niemann-Pick disease, type B, 607616	YES	NO	NO	YES	NO
0	ENSG00000166333	-	-	-	YES	YES	YES

0	ENSG00000166337	-	-	-	YES	NO	YES
0	ENSG00000166340	-	-	-	NO	YES	YES
0	ENSG00000166441	-	-	-	YES	NO	YES
0	ENSG00000166454	-	-	-	YES	NO	YES
0	ENSG00000166483	-	-	-	YES	YES	YES
0	ENSG00000166484	-	-	-	YES	YES	YES
0	ENSG00000166501	-	-	-	YES	YES	NO
0	ENSG00000166508	-	-	-	YES	NO	YES
0	ENSG00000166598	-	-	-	YES	NO	YES
0	ENSG00000166813	-	-	-	YES	NO	YES
0	ENSG00000166851	-	-	-	YES	YES	YES
0	ENSG00000166949	-	-	-	YES	YES	NO
0	ENSG00000167004	-	-	-	NO	YES	YES
0	ENSG00000167085	-	-	-	YES	NO	YES
0	ENSG00000167114	-	-	-	NO	YES	YES
0	ENSG00000167136	-	-	-	YES	YES	YES
0	ENSG00000167182	-	-	-	YES	NO	YES
0	ENSG00000167193	-	-	-	YES	NO	YES
0	ENSG00000167244	-	-	-	YES	YES	YES
0	ENSG00000167461	-	-	-	YES	YES	NO
0	ENSG00000167468	-	-	-	NO	YES	YES
0	ENSG00000167491	-	-	-	NO	YES	YES
0	ENSG00000167658	-	-	-	YES	YES	NO
0	ENSG00000167670	-	-	-	YES	NO	YES
0	ENSG00000167972	-	-	-	NO	YES	YES
0	ENSG00000167986	-	-	-	NO	YES	YES
0	ENSG00000168003	-	-	-	YES	NO	YES
0	ENSG00000168036	-	-	-	YES	YES	YES
0	ENSG00000168090	-	-	-	YES	NO	YES
0	ENSG00000168282	Carbohydrate-deficient glycoprotein syndrome, type II, 212066	YES	NO	NO	NO	YES
0	ENSG00000168283	-	-	-	YES	NO	YES
0	ENSG00000168348	-	-	-	YES	YES	NO
0	ENSG00000168610	-	-	-	YES	YES	YES
0	ENSG00000168621	-	-	-	YES	NO	YES
0	ENSG00000169032	-	-	-	YES	YES	YES
0	ENSG00000169047	-	-	-	YES	NO	YES
0	ENSG00000169071	-	-	-	YES	NO	YES
0	ENSG00000169083	Prostate cancer; Perineal hypospadias; Androgen insensitivity, several forms, 300068; Infertile male syndrome, 308370; Spinal and bulbar muscular atrophy of Kennedy, 313200; Breast cancer, male, with Reifenstein syndrome,	NO	YES	NO	YES	NO

		312300					
0	ENSG00000169297	Adrenal hypoplasia, congenital, with hypogonadotropic hypogonadism, 300200; Dosage-sensitive sex reversal, 300018	NO	YES	YES	YES	NO
0	ENSG00000169398	-	-	-	YES	YES	YES
0	ENSG00000169410	-	-	-	YES	NO	YES
0	ENSG00000169679	-	-	-	YES	NO	YES
0	ENSG00000169710	-	-	-	YES	NO	YES
0	ENSG00000169756	-	-	-	YES	NO	YES
0	ENSG00000169884	-	-	-	YES	YES	NO
0	ENSG00000169919	-	-	-	YES	YES	YES
0	ENSG00000169946	-	-	-	NO	YES	YES
0	ENSG00000170312	-	-	-	NO	YES	YES
0	ENSG00000170345	-	-	-	YES	YES	YES
0	ENSG00000170365	-	-	-	NO	YES	YES
0	ENSG00000170385	-	-	-	NO	YES	YES
0	ENSG00000170421	-	-	-	NO	YES	YES
0	ENSG00000170558	-	-	-	YES	YES	YES
0	ENSG00000170820	Ovarian dysgenesis, hypergonadotropic, with normal karyotype, 233300	NO	YES	NO	NO	YES
0	ENSG00000170836	-	-	-	YES	NO	YES
0	ENSG00000170927	-	-	-	NO	YES	YES
0	ENSG00000170961	-	-	-	NO	YES	YES
0	ENSG00000170989	-	-	-	YES	YES	YES
0	ENSG00000171100	Myotubular myopathy 1, 310400	YES	NO	NO	YES	YES
0	ENSG00000171105	Leprechaunism, 246200; Noninsulin-dependent diabetes mellitus, 125853; Rabson-Mendenhall syndrome, 262190; Diabetes mellitus, insulin-resistant, with acanthosis nigricans, 610549; Familial hyperinsulinemic hypoglycemia-5, 609968	YES	NO	NO	YES	YES
0	ENSG00000171298	Glycogen storage disease II, 232300	YES	NO	NO	YES	NO
0	ENSG00000171316	-	-	-	NO	YES	YES
0	ENSG00000171345	-	-	-	YES	NO	YES
0	ENSG00000171522	-	-	-	NO	YES	YES
0	ENSG00000171552	-	-	-	YES	YES	YES
0	ENSG00000171587	-	-	-	YES	NO	YES
0	ENSG00000171720	-	-	-	YES	YES	YES
0	ENSG00000171723	Molybdenum cofactor deficiency, 252150	YES	NO	NO	NO	YES
0	ENSG00000171791	-	-	-	YES	YES	YES
0	ENSG00000171862	-	-	-	YES	YES	YES
0	ENSG00000171956	-	-	-	YES	NO	YES
0	ENSG00000172071	Wolcott-Rallison syndrome, 226980	YES	NO	NO	NO	YES
0	ENSG00000172270	-	-	-	NO	YES	YES
0	ENSG00000172273	-	-	-	YES	NO	YES
0	ENSG00000172482	Hyperoxaluria, primary, type 1, 259900	YES	NO	NO	YES	NO

0	ENSG00000172638	-	-	-	NO	YES	YES
0	ENSG00000172732	-	-	-	NO	YES	YES
0	ENSG00000172809	-	-	-	YES	NO	YES
0	ENSG00000172893	Smith-Lemli-Opitz syndrome, type I, 270400; Smith-Lemli-Opitz syndrome, type II, 268670	YES	NO	NO	YES	YES
0	ENSG00000172939	-	-	-	YES	NO	YES
0	ENSG00000172977	-	-	-	NO	YES	YES
0	ENSG00000173020	-	-	-	YES	NO	YES
0	ENSG00000173039	-	-	-	NO	YES	YES
0	ENSG00000173402	-	-	-	NO	YES	YES
0	ENSG00000173653	-	-	-	NO	YES	YES
0	ENSG00000173801	-	-	-	YES	NO	YES
0	ENSG00000173812	-	-	-	YES	YES	NO
0	ENSG00000174238	-	-	-	YES	NO	YES
0	ENSG00000174405	-	-	-	NO	YES	YES
0	ENSG00000174437	-	-	-	YES	NO	YES
0	ENSG00000175054	-	-	-	NO	YES	YES
0	ENSG00000175104	-	-	-	NO	YES	YES
0	ENSG00000175198	Propionic acidemia, type I or pccA type, 606054	YES	NO	NO	YES	YES
0	ENSG00000175305	-	-	-	YES	YES	NO
0	ENSG00000175354	-	-	-	YES	YES	NO
0	ENSG00000175387	-	-	-	YES	YES	YES
0	ENSG00000175445	-	-	-	NO	YES	YES
0	ENSG00000175592	-	-	-	YES	NO	YES
0	ENSG00000175595	-	-	-	YES	NO	YES
0	ENSG00000175745	-	-	-	YES	NO	YES
0	ENSG00000175920	-	-	-	NO	YES	YES
0	ENSG00000176165	-	-	-	NO	YES	YES
0	ENSG00000176208	-	-	-	NO	YES	YES
0	ENSG00000176225	-	-	-	YES	NO	YES
0	ENSG00000176248	-	-	-	YES	NO	YES
0	ENSG00000176619	-	-	-	YES	NO	YES
0	ENSG00000176697	-	-	-	NO	YES	YES
0	ENSG00000176890	-	-	-	YES	YES	NO
0	ENSG00000177302	-	-	-	NO	YES	YES
0	ENSG00000177426	Holoprosencephaly-4, 142946	YES	NO	NO	NO	YES
0	ENSG00000177463	-	-	-	YES	NO	YES
0	ENSG00000177606	-	-	-	YES	YES	YES
0	ENSG00000177731	-	-	-	NO	YES	YES
0	ENSG00000177885	-	-	-	YES	NO	YES
0	ENSG00000178028	-	-	-	YES	NO	YES
0	ENSG00000178209	-	-	-	NO	YES	YES
0	ENSG00000178537	-	-	-	YES	YES	NO
0	ENSG00000178568	-	-	-	YES	NO	YES
0	ENSG00000178585	-	-	-	YES	NO	YES

0	ENSG00000178607	-	-	-	YES	YES	YES
0	ENSG00000178691	-	-	-	YES	NO	YES
0	ENSG00000178726	-	-	-	NO	YES	YES
0	ENSG00000178802	Carbohydrate-deficient glycoprotein syndrome, type Ib, 602579	YES	NO	NO	NO	YES
0	ENSG00000178951	-	-	-	NO	YES	YES
0	ENSG00000178966	-	-	-	YES	NO	YES
0	ENSG00000178999	-	-	-	YES	YES	YES
0	ENSG00000179218	-	-	-	NO	YES	YES
0	ENSG00000179295	-	-	-	YES	NO	YES
0	ENSG00000179348	-	-	-	NO	YES	YES
0	ENSG00000180176	-	-	-	NO	YES	YES
0	ENSG00000180209	-	-	-	YES	NO	YES
0	ENSG00000180210	-	-	-	NO	YES	YES
0	ENSG00000180370	-	-	-	YES	YES	YES
0	ENSG00000180447	-	-	-	YES	NO	YES
0	ENSG00000180900	-	-	-	NO	YES	YES
0	ENSG00000181027	-	-	-	NO	YES	YES
0	ENSG00000181090	-	-	-	YES	NO	YES
0	ENSG00000181104	-	-	-	YES	NO	YES
0	ENSG00000181222	-	-	-	YES	NO	YES
0	ENSG00000181915	-	-	-	YES	YES	NO
0	ENSG00000181965	-	-	-	YES	NO	YES
0	ENSG00000182263	-	-	-	YES	NO	YES
0	ENSG00000182511	-	-	-	YES	YES	YES
0	ENSG00000182578	-	-	-	YES	NO	YES
0	ENSG00000182621	-	-	-	YES	NO	YES
0	ENSG00000182742	-	-	-	YES	NO	YES
0	ENSG00000182963	-	-	-	NO	YES	YES
0	ENSG00000183023	-	-	-	YES	YES	YES
0	ENSG00000183072	-	-	-	NO	YES	YES
0	ENSG00000183337	-	-	-	NO	YES	YES
0	ENSG00000183495	-	-	-	YES	NO	YES
0	ENSG00000183691	-	-	-	YES	NO	YES
0	ENSG00000183735	-	-	-	YES	NO	YES
0	ENSG00000183770	Blepharophimosis, ptosis, and epicanthus inversus syndrome, 110100	NO	YES	NO	NO	YES
0	ENSG00000183853	-	-	-	NO	YES	YES
0	ENSG00000183873	-	-	-	NO	YES	YES
0	ENSG00000184058	-	-	-	NO	YES	YES
0	ENSG00000184304	-	-	-	YES	YES	YES
0	ENSG00000184349	-	-	-	YES	NO	YES
0	ENSG00000184432	-	-	-	YES	YES	NO
0	ENSG00000184470	-	-	-	NO	YES	YES
0	ENSG00000184557	-	-	-	YES	YES	YES
0	ENSG00000184916	-	-	-	YES	NO	YES

0	ENSG00000184937	Frasier syndrome, 136680; Denys-Drash syndrome, 194080	NO	YES	YES	YES	YES
0	ENSG00000185010	Hemophilia A, 134500	YES	NO	YES	YES	NO
0	ENSG00000185122	-	-	-	NO	YES	YES
0	ENSG00000185338	-	-	-	NO	YES	YES
0	ENSG00000185532	-	-	-	YES	NO	YES
0	ENSG00000185559	-	-	-	NO	YES	YES
0	ENSG00000185650	-	-	-	YES	NO	YES
0	ENSG00000185787	-	-	-	YES	YES	YES
0	ENSG00000185883	-	-	-	YES	NO	YES
0	ENSG00000185920	-	-	-	YES	NO	YES
0	ENSG00000186111	-	-	-	YES	NO	YES
0	ENSG00000186153	-	-	-	YES	YES	YES
0	ENSG00000186260	-	-	-	NO	YES	YES
0	ENSG00000186575	-	-	-	NO	YES	YES
0	ENSG00000186810	-	-	-	YES	YES	NO
0	ENSG00000186895	-	-	-	YES	NO	YES
0	ENSG00000187098	-	-	-	NO	YES	YES
0	ENSG00000187323	-	-	-	YES	NO	YES
0	ENSG00000187391	-	-	-	YES	NO	YES
0	ENSG00000187714	-	-	-	NO	YES	YES
0	ENSG00000187735	-	-	-	YES	NO	YES
0	ENSG00000187908	Glioblastoma multiforme, 137800; Medulloblastoma, 155255	YES	NO	NO	NO	YES
0	ENSG00000188157	-	-	-	NO	YES	YES
0	ENSG00000188566	-	-	-	YES	YES	NO
0	ENSG00000188641	-	-	-	YES	YES	NO
0	ENSG00000188910	-	-	-	NO	YES	YES
0	ENSG00000188986	-	-	-	YES	NO	YES
0	ENSG00000196218	-	-	-	NO	YES	YES
0	ENSG00000196284	-	-	-	YES	YES	NO
0	ENSG00000196367	-	-	-	YES	NO	YES
0	ENSG00000196419	-	-	-	YES	YES	NO
0	ENSG00000196468	-	-	-	YES	NO	YES
0	ENSG00000196588	-	-	-	YES	NO	YES
0	ENSG00000196591	-	-	-	YES	NO	YES
0	ENSG00000196628	-	-	-	YES	NO	YES
0	ENSG00000196712	-	-	-	NO	YES	YES
0	ENSG00000196839	Severe combined immunodeficiency due to ADA deficiency, 102700	YES	NO	NO	YES	YES
0	ENSG00000196878	-	-	-	NO	YES	YES
0	ENSG00000197063	-	-	-	YES	NO	YES
0	ENSG00000197081	-	-	-	YES	NO	YES
0	ENSG00000197102	-	-	-	NO	YES	YES
0	ENSG00000197122	-	-	-	NO	YES	YES
0	ENSG00000197283	-	-	-	YES	YES	YES

0	ENSG00000197323	-	-	-	YES	NO	YES
0	ENSG00000197381	-	-	-	NO	YES	YES
0	ENSG00000197386	-	-	-	NO	YES	YES
0	ENSG00000197442	-	-	-	YES	YES	NO
0	ENSG00000197461	-	-	-	NO	YES	YES
0	ENSG00000197535	-	-	-	NO	YES	YES
0	ENSG00000197616	-	-	-	NO	YES	YES
0	ENSG00000197746	Metachromatic leukodystrophy due to deficiency of SAP-1, 249900; Gaucher disease, variant form, 176801	YES	NO	NO	YES	YES
0	ENSG00000198001	-	-	-	YES	YES	NO
0	ENSG00000198055	-	-	-	YES	YES	NO
0	ENSG00000198400	-	-	-	NO	YES	YES
0	ENSG00000198431	-	-	-	NO	YES	YES
0	ENSG00000198625	-	-	-	YES	YES	YES
0	ENSG00000198626	-	-	-	NO	YES	YES
0	ENSG00000198646	-	-	-	YES	YES	YES
0	ENSG00000198668	-	-	-	YES	YES	NO
0	ENSG00000198707	-	-	-	YES	YES	NO
0	ENSG00000198719	-	-	-	YES	YES	YES
0	ENSG00000198743	-	-	-	NO	YES	YES
0	ENSG00000198793	-	-	-	YES	YES	YES
0	ENSG00000198805	-	-	-	NO	YES	YES
0	ENSG00000198807	Oligodontia, 604625	YES	NO	NO	NO	YES
0	ENSG00000198814	-	-	-	YES	YES	YES
0	ENSG00000198836	-	-	-	NO	YES	YES
0	ENSG00000198900	-	-	-	NO	YES	YES
0	ENSG00000198909	-	-	-	YES	YES	YES
0	ENSG00000198917	-	-	-	YES	NO	YES
0	ENSG00000198918	-	-	-	YES	YES	NO
0	ENSG00000198931	-	-	-	NO	YES	YES
0	ENSG00000198947	-	-	-	NO	YES	YES
0	ENSG00000204103	-	-	-	NO	YES	YES
0	ENSG00000204217	-	-	-	YES	NO	YES
0	ENSG00000204262	-	-	-	YES	NO	YES
0	ENSG00000204370	-	-	-	YES	NO	YES
0	ENSG00000204628	-	-	-	YES	YES	NO
0	ENSG00000205560	-	-	-	YES	NO	YES
0	ENSG00000205581	-	-	-	YES	NO	YES
0	ENSG00000213341	-	-	-	YES	NO	YES
0	ENSG00000213585	-	-	-	NO	YES	YES
0	ENSG00000213930	Galactosemia, 230400	YES	YES	NO	YES	NO
0	ENSG00000214367	-	-	-	YES	YES	NO
0	ENSG00000214517	-	-	-	YES	NO	YES
0	ENSG00000221818	-	-	-	YES	NO	YES
0	ENSG00000221823	-	-	-	YES	NO	YES

0	ENSG00000221983	-	-	-	YES	YES	NO
