# Supplementary material for: New genes drive the evolution of gene interaction networks in the human and mouse genomes
Source: Genome Biol. 2015 Oct 1;16:202. doi: 10.1186/s13059-015-0772-4 (PMC4590697; doi:10.1186/s13059-015-0772-4)
Supplement: Additional file 8: Table S4. — Characteristics of network topology and brain expression pattern for human lineage-specific hub genes. (PDF 7 kb) [file 13059_2015_772_MOESM8_ESM.pdf]

Table S4: Characteristics of network topology and brain expression pattern for human lineage-specific hub genes

Ensembl ID	Gene Symbol	Phylogenetic Branch	PPI Connectivity	Brain Expression Pattern
ENSG00000154608	CEP170P1	12	16	Fetus brain biased
ENSG00000145002	FAM86B2	12	15	Fetus brain biased
ENSG00000155428	TRIM74	12	41	Brain unbiased
ENSG00000212829	RPS26P11	12	6	Brain unbiased
