# Supplementary material for: New genes drive the evolution of gene interaction networks in the human and mouse genomes
Source: Genome Biol. 2015 Oct 1;16:202. doi: 10.1186/s13059-015-0772-4 (PMC4590697; doi:10.1186/s13059-015-0772-4)
Supplement: Additional file 9: Table S5. — Summary of brain-function related genes with literature evidence. (PDF 97 kb) [file 13059_2015_772_MOESM9_ESM.pdf]

Table S5: Summary of brain-function related genes with literature evidence

Entrez Gene ID	Gene Symbol	Experimental organism(s)	Evidence description	Reference (PMID)
862	RUNX1T1	Rat	RUNX1T1 has highest expression level in brain relative to other organs; Decreased Runx1t1 expression reduced the neuronal differentiation of RGCs, while increased Runx1t1 expression caused a greater number of RGCs to differentiate into neurons.	25473084
55885	LMO3	Mouse	LMO3 is a transcriptional regulator involved in central nervous system development and neuroblastoma.	25176312
51225	ABI3	Rat	ABI3 is one antibody of NT1 neurotensin receptors, and immunoblot experiments that used Abi3 revealed the presence of two differentially glycosylated forms of the NT1 receptor in developing rat brain.	10940941
138046	RALYL	Human	RALYL is expressed in human fetus brain, and was shown to interact with LRRK2, pathogenesis of Parkinson's disease, and other neurodegerations	19001729
9859	CEP170	Human	CEP170 was implicated as a novel genecausative for corpus callosal abnormalities (CCA) in patients with a terminal 1q deletion.	21934713
23357	SNAPIN	Human	Snapin deficiency is associated with developmental defects of the central nervous system.	20946101
7965	AIMP2	Human	AIMP2 was reported to be involved in the development of Parkinson's disease.	24786396
5269	SERPINB6	Mouse	Serpinb6 was claimed as an endogenous inhibitor of neuropsin in brain.	12638128
57596	BEGAIN	Rat	BEGAIN was shown to be specifically expressed in brain and enriched in the PSD fraction.	9756850
3801	KIFC3	Human; Mouse	Disturbances of KIF3 could cause the dysregulation of retrograde axonal transports, which would leads to motor neuron degeneration.	23006449
10381	TUBB3	Human	Genetic variations affecting all beta-tubulin genes expressed at high levels in the brain (TUBB2B, TUBB3, TUBB, TUBB4A, and TUBB2A) have been linked with malformations of cortical development.	24702957
7280	TUBB2A	Human	As above	24702957
10382	TUBB4A	Human	As above	24702957
10576	CCT2	Rat	The protein expression of CCT2 was detected in rat brain.	20049628
10575	CCT4	Human	CCT4 was reported to be associated with Alzheimer's.	22482075
10694	CCT8		CCT8 was reported to be associated with Down syndrome.	
6950	TCP1	Mouse	Tcp1 was found to be associated with high-affinity choline uptake (HACU) in the mouse brain	7665164
7314	UBB	Human	UBB was found to be associated with hypothalamic neurodegeneratio.	22285186
222484	LNX2	Mouse	The protein expression of LNX2 was detected in brain tissue.	25200495
115992	RNF166	Mouse	RNF166 was shown to be target gene of TRα1 (thyroid hormone receptor α1), which is well recognized for its importance in brain development	23398480

9958	USP15	Human	USP15 was clearly found to be expressed in brain tissue.	24852371
7681	MKRN3	Mouse	Mkrn3 was found preferentially expressed in specific brain regions in mouse.	12971993
648	BMI1	Human; Mouse	Bmi1 is down-regulated in the aging brain and displays antioxidant and protective activities in neurons.	22384090
672	BRCA1	Human; Mouse	Apoptotic and centrosomal functions of BRCA1 in neural progenitors was indicated to be involved in the embryonic brain to DNA damage, as well as the developmental regulation of brain size	24639535
7251	TSG101	Mouse	Abnormal regulation of TSG101 was found to be associated with spongiform neurodegeneration in mouse.	19703557
9099	USP2	Mouse	Using multiple methods, USP2 expression was clearly observed mouse brain.	14686789
9921	RNF10	Mouse	RNF10 was found to be deregulated in brain samples of a FXTAS mouse model.	24418349
51529	ANAPC11	Human	Through Northern blot hybridization, ANAPC11 was detected in brain with moderate signal.	11573242
55743	CHFR	Human	Chfr is frequently methylated in cell lines derived from tumors of brain (100%).	12538348
8454	CUL1	Human	Cul1 was reported to be involved in the pathogenesis of human glioma, a kind of brain tumor.	25201578
5885	RAD21	Human	The co-ordinated expression of separase, securin and Rad21 is fundamental for the developing brain.	17450531
9768	KIAA0101	Mouse	It was reported the major sites of expression of this gene during mouse embryogenesis include the facial prominences, limbs, somites, brain, spinal cord and hair follicles.	16815099
