# Supplementary material for: New genes drive the evolution of gene interaction networks in the human and mouse genomes
Source: Genome Biol. 2015 Oct 1;16:202. doi: 10.1186/s13059-015-0772-4 (PMC4590697; doi:10.1186/s13059-015-0772-4)
Supplement: Additional file 10: Figure S5. — Distribution of young genes (primate-specific genes) that originated from duplication-based and de novo mechanisms. (PDF 48 kb) [file 13059_2015_772_MOESM10_ESM.pdf]

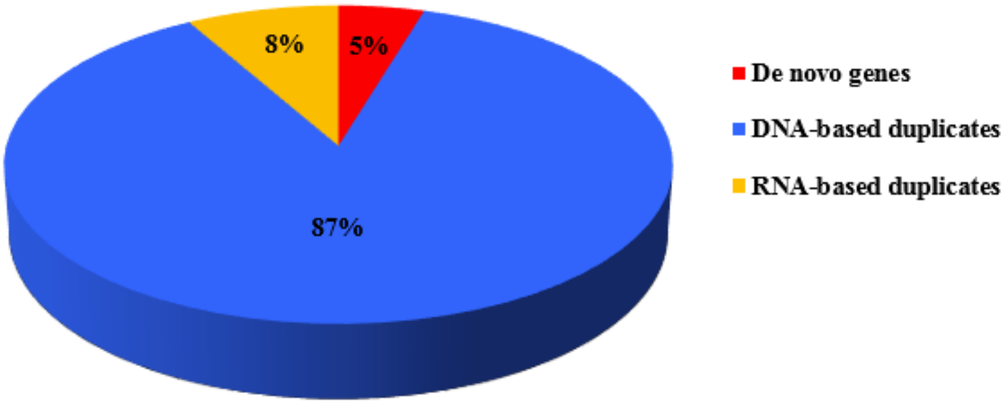

Figure S5 Distribution of young genes (primate-specific genes) that originated from duplication-based and de novo mechanisms.
