# Supplementary material for: New genes drive the evolution of gene interaction networks in the human and mouse genomes
Source: Genome Biol. 2015 Oct 1;16:202. doi: 10.1186/s13059-015-0772-4 (PMC4590697; doi:10.1186/s13059-015-0772-4)
Supplement: Additional file 11: Figure S6. — Comparison of gene features between young genes (primate-specific) and old genes. (PDF 114 kb) [file 13059_2015_772_MOESM11_ESM.pdf]

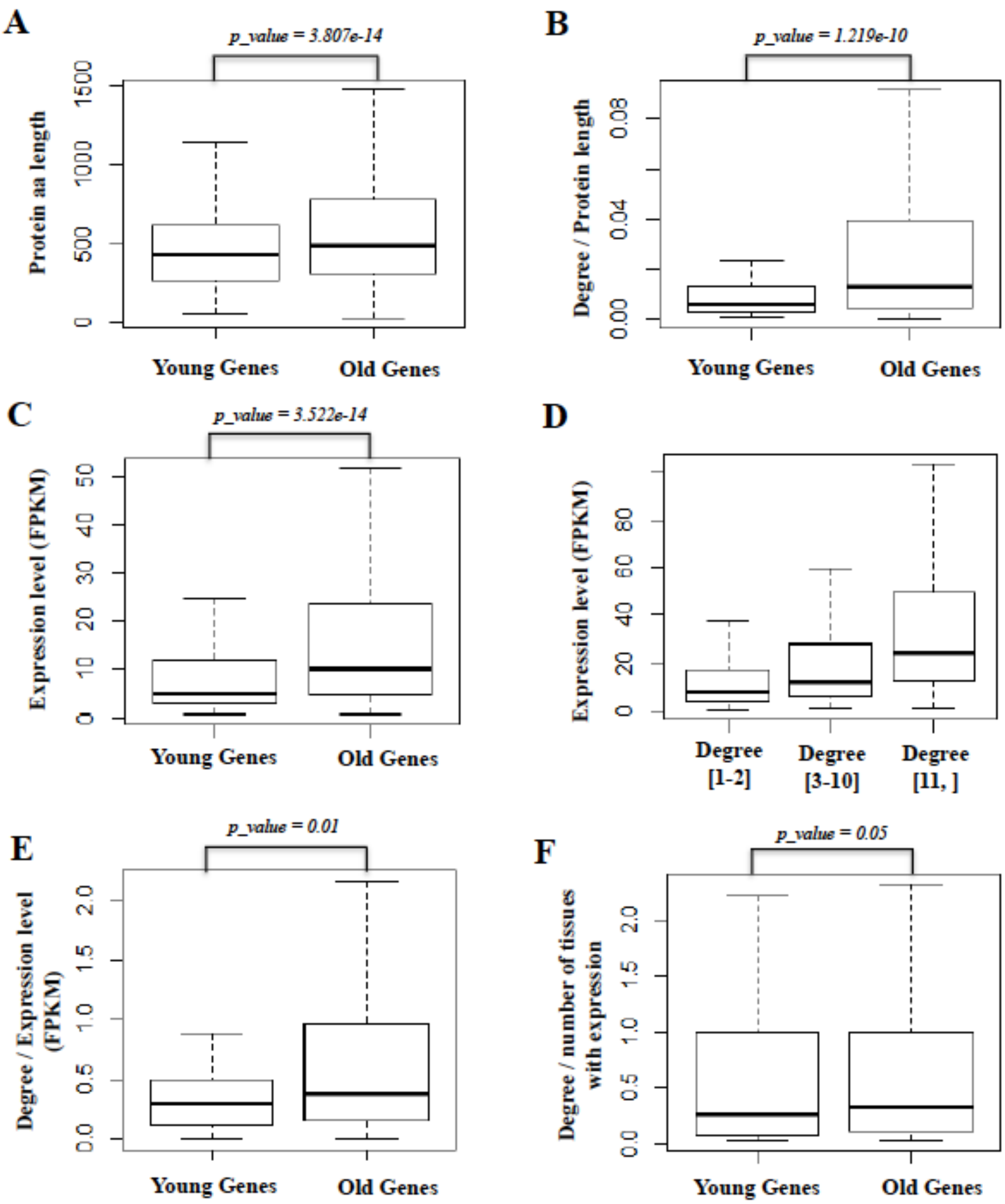

Figure S6 Comparison of the features between young genes (primate-specific) and old genes. (A) Comparison of protein lengths between young genes and old genes. (B) Comparison of normalized PPI degree between young genes and old genes. Normalized PPI degree was defined as PPI degree for each gene divided by its coded protein length. (C) Comparison of gene expression between young genes (primate-specific) and old genes. (D) Positive correlation between PPI network degree and gene expression levels. (E) Comparison of normalized PPI degree of expression level between young genes and old genes. Normalized PPI degree of expression level was defined as PPI degree for each gene divided by its average expression level in all the tissues with its expression (FPKM >=1). (F) Comparison of normalized PPI degree of expression breadth between young genes and old genes. Normalized PPI degree of expression breadth was defined as PPI degree for each gene divided by the number of tissue with expression (FPKM >=1). The statistic p_value was computed with Wilcoxon signed-rank test.
