# Supplementary material for: New genes drive the evolution of gene interaction networks in the human and mouse genomes
Source: Genome Biol. 2015 Oct 1;16:202. doi: 10.1186/s13059-015-0772-4 (PMC4590697; doi:10.1186/s13059-015-0772-4)
Supplement: Additional file 12: Table S6. — Protein sequence features of young hubs and young non-hubs. (PDF 10 kb) [file 13059_2015_772_MOESM12_ESM.pdf]

Table S6: Protein sequence features of young hubs and young non-hubs.

Category	Protein Length (Median)	Low Complexity (%, Median)	Long_Disorder (%, Median)	Short_Disorder (%, Median)
**Hubs**	395	6.26%	13.67%	14.62%
**Non-hubs**	436.5	4.12%	6.99%	10.47%

Note: Genes (proteins) with minimum interaction degree 6 (medium connectivity) were defined as hubs, the remaining nodes as non-hubs.
